# Supplementary material for: A scalable reinforcement learning approach for screening large peptide libraries for bioactive peptide discovery
Source: Nat Commun. 2025 Nov 27;16:11685. doi: 10.1038/s41467-025-66748-y (PMC12748989; doi:10.1038/s41467-025-66748-y)
Supplement: Supplementary file 1 — Supplementary Information [file 41467_2025_66748_MOESM1_ESM.pdf]

`

A Scalable Reinforcement Learning Approach for Screening Large Peptide Libraries for Bioactive Peptide Discovery

Supplementary Information

Mohit Pandey[1,2], Jane Foo[1,2†], Shabnam Massah[1,2†], Morgan A Alford[3†], Hazem Mslati[4†], Gopeshh Subbaraj[5], Mira Saba[1], Francesco Gentile[4,6], Nada Lallous[1,2], Evan F Haney[3], Robert E. W. Hancock[3], Martin Ester[7], Artem Cherkasov[1,2]*

[1] Vancouver Prostate Centre, University of British Columbia, Vancouver, British Columbia, Canada
[2]Faculty of Medicine, University of British Columbia, Vancouver, BC, Canada
[3]Centre for Microbial Diseases and Immunity Research, Department of Microbiology and Immunology, University of British Columbia, Vancouver, BC, Canada
[4] Department of Chemistry and Biomolecular Sciences, University of Ottawa, Ontario, Canada
[5]Mila, Université de Montréal, QC, Canada
[6] Ottawa Institute of Systems Biology, Ottawa, Ontario, Canada
[7]School of Computing Science, Simon Fraser University, Burnaby, BC, Canada

*Corresponding author(s). E-mail(s): acherkasov@prostatecentre.com; †These authors contributed equally to this work.

`

Supplementary Note 1: PepSce Workflow

The role of helicity in ACPs

The proposed approach to discovering potent 12-mer ACPs using the PepSce workflow is guided by the structural and functional properties inherent to the helical secondary structure of peptides. These peptides are characterized by their amphipathic nature, possessing distinct polar (hydrophilic) and non-polar (hydrophobic) regions that enable them to integrate compatibly into the lipid bilayers of cancer cell membranes, which are often enriched with negatively charged phospholipids like 1-palmitoyl-2-oleoyl-glycero-3-phosphatidylserine (PS)[1]. It is observed that the integration of positively charged residues, such as lysine and arginine, into the peptides sequence enhances electrostatic interactions with these membranes which triggers membrane disruption[1,2]. Furthermore, the helical structure of these ACPs not only maximizes their contact area with the cell membrane but also plays a crucial role in their ability to induce either necrotic or apoptotic cell death[3]. Additionally, the distinctive properties of cancer cell membranes, such as increased fluidity and an abundance of microvilli, boost the probability of peptide interactions, thereby enhancing the specificity of ACPs towards cancer cells and improving their therapeutic efficacy[2]. In this work, the secondary structure analysis for dataset curation was performed using the DSSP module of biopython library[4].

Evaluating Helicity Assumptions in PDB-Derived Peptides

The PDB is inherently biased toward soluble (non-membrane) protein structures, and short polypeptides exhibit significant conformational plasticity, with their folding behavior highly dependent on the surrounding environment. Consequently, no peptide sequence is intrinsically helical across all conditions. In this study, the assumption of helicity in selected peptides was a necessary simplification to guide the ACP discovery pipeline. Rather than presuming that PDB-derived peptides maintain a helical conformation universally, we prioritized them for their evolutionary origins and potential functional relevance in biological systems. Many membranolytic peptides, including Mastoparan, exist as random coils in aqueous buffers but adopt helical conformations upon interacting with membrane environments[5–9]. Our focus is on the peptide's structure at this biologically relevant time point—when it engages with the membrane—rather than in intermediary, unstructured states.

Beyond relying on static helical annotations from DSSP, we incorporated physicochemical descriptors that remain largely invariant to environmental conditions, such as amino acid composition, molecular weight, and charge distribution. To further assess the validity of our helicity assumption, we retrospectively analyzed 20 randomly selected peptides from the 95 PDB-derived sequences that underwent experimental validation. Of these, 15 displayed a predicted helicity of over 60% with an overall confidence of 70%, as determined by an expert jury comprising four secondary structure prediction tools—PSIPRED[10], JNET[11], RANSSEC (a locally

`

developed tool by the authors of PROTEUS), and structural alignment (XALIGN)[12]. Prior literature has established helicity thresholds between 30–50% to classify structures as predominantly helical[13–15]. For the five peptides where the expert jury did not reach consensus, we conducted additional context-dependent secondary structure predictions using state-of-the-art methods such as AlphaFold3[16] and JPred[17], which assess folding tendencies across diverse environments. Both AlphaFold3 and JPred consistently predicted these peptides to be helical with high confidence. These results are available in Supplementary File 2 (helicity.doc).

Rationale for Selecting 12-Mer Peptides

The selection of 12-mers as the target peptide length was driven by considerations related to high-throughput synthesis, exploration of novel chemical space, and practical feasibility for validation. The spot-array synthesis method used for experimental validation requires uniform peptide lengths within the same array, necessitating a fixed-length design. A length of 12 residues was chosen based on practical synthesis constraints and cost considerations, ensuring efficient and scalable peptide production.

Additionally, the training dataset for the oracle regression model was derived from Mastoparan analogues, which were 14-mers. To extend the chemical space beyond the training distribution while maintaining structural relevance, 12-mers were selected. This length preserves key amphipathic helical features associated with membranolytic ACPs while providing sufficient variation from the training sequences to enable broader exploration.

From a synthetic and validation perspective, peptides in the 10–15 residue range generally exhibit favorable synthetic yields and cost-effectiveness. The selection of 12-mers represents a balance between structural complexity and practical feasibility, making them an optimal choice for initial discovery while allowing room for further sequence refinement and optimization.

Challenges in Using 3D/4D Descriptors for Large-Scale Peptide Screening

While 3D and 4D descriptors are highly effective for studying peptide structure-activity relationships at a fine level of detail, their application in large-scale virtual screening presents several practical challenges.

One major limitation is computational complexity. Unlike small molecules, which often adopt stable low-energy conformations, peptides exist as dynamic ensembles, constantly interconverting in solution. Generating accurate 3D conformers requires computationally expensive methods such as molecular dynamics (MD), enhanced sampling techniques, or quantum mechanics-based optimizations. The challenge is even greater for 4D descriptors, which involve conformational profiling over time and across different environmental conditions.

Scalability is another concern. The computational cost of generating 3D/4D descriptors increases exponentially with dataset size. In our study, we evaluated millions of candidate peptides, making exhaustive conformational sampling infeasible. As shown in Figure 3A, 3D-based inductive descriptors perform reasonably well in cell inhibition prediction for MDA-MB-231 cell lines,

`

however our analysis estimates that computing these 3D-based inductive descriptors for a single iteration of our screening pipeline would take nearly 68 years for 36 million peptides, assuming no parallelization (Supplementary Table 1). Even with high-performance computing (HPC) resources, this remains a major bottleneck, making 3D/4D-based screening impractical for high-throughput virtual screening.

Given these constraints, we rely on 2D descriptors, which provide a more scalable and empirically validated approach. Prior research has shown that 2D sequence-derived and physicochemical descriptors—such as hydrophobicity, charge, amphipathicity, and residue composition—offer strong predictive power for peptide activity in machine learning-based models. Studies in antimicrobial and anticancer peptide discovery (e.g., DeepAmPep30[18], Das et al.[19], xdeepACP[20], etc.) have successfully built predictive frameworks using 2D descriptors alone, without requiring full 3D structural characterization.

Supplementary Note 2: TARSA Baselines

Due to the scarcity of literature on DL-based screening of ACP libraries, the choice of baselines was guided by traditional bioactive peptide discovery principles. These include (i) exhaustive ranking and filtering of candidates, and (ii) local similarity search based on known bioactives. The screening ability of TARSA was also compared for GEN_{12mer} dataset generated by naïve probabilistic sampling of amino acids from helical peptide distribution with other data prudent generative paradigms[21,22].

Here, more details on our baseline experiments are provided. In order to ensure fair comparison between screening and generative models, we limit our benchmarking to 50,000 samples from each approach.

QSAR screening with oracle proxy

This corresponds to brute force ranking of all peptides in the library using a trained surrogate function (oracle proxy) for bioactivity prediction. Here we use the same oracle proxy as the one driving r_{oracle} of the TARSA reward function. As seen in Supplementary Table 1, the enumeration and feature extraction of a large number of peptides can be extremely expensive, thereby making a brute force ranking and screening of large libraries time consuming and possibly computationally infeasible with reasonable computational budgets. Three independent instances of 50,000 peptides were randomly sampled from the PDB-12mer dataset and the metrics for top-100 and first-100 sampled peptides from each instance were reported.

Supplementary Table 1 | Projected time to compute the descriptors for 36M peptides from PDB-large dataset on a single CPU machine. For each of the descriptors, observed peptide count is the number of samples for which we computed that descriptor. The time estimates were linearly extrapolated to get the projected time for the library.

`

**Descriptor**	**Projected Time**	**Observed peptide Count**
Modlamp	2.1 years	500000
ESM	1.5 days	2000000
AutoEncoder	2 days	2000000
Inductive	68.5 years	1000
ifeature	1.9 days	500000
BOWPE	12 hours	2000000

Local similarity search

Local similarity search attempts to identify regions of similarity between two sequences, thereby discovering peptides with similar functional or structural properties. Its speed enables quick search through large databases hence making them suitable for screening of large peptide libraries for potential candidates. Typically, these algorithms operate by searching the peptide database against a query sequence (i.e. usually a known bioactive). In this study, instead of just one query sequence, the entire $D_{mastoparan}$ was utilized to find the most similar peptides. $D_{mastoparan}$ was projected to the same lower dimensional representations as used by TARSA (ESM projections). Next, the convex hull spanned by the peptides in $D_{mastoparan}$ was computed in this low dimensional space (Supplementary Fig 1). For the peptides contained within the convex hull, the oracle proxy was queried and peptides were ranked.

`

A.

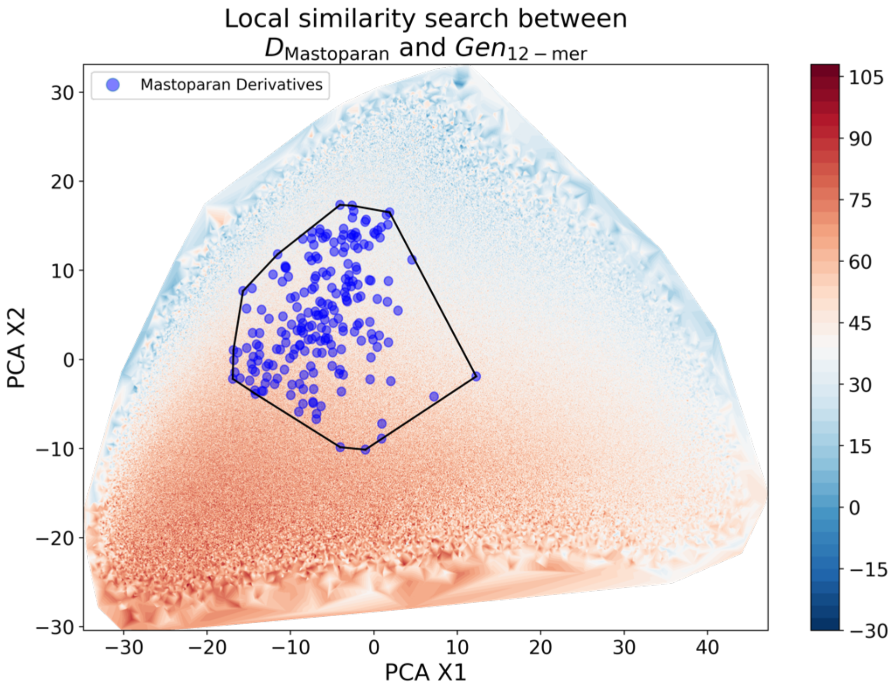

Local similarity search between
$D_{\mathrm{Mastoparan}}$ and $Gen_{12-\mathrm{mer}}$
Mastoparan Derivatives
PCA X2
PCA X1
30
20
10
0
−10
−20
−30
−30
−20
−10
0
10
20
30
40
105
90
75
60
45
30
15
0
−15
−30

B.

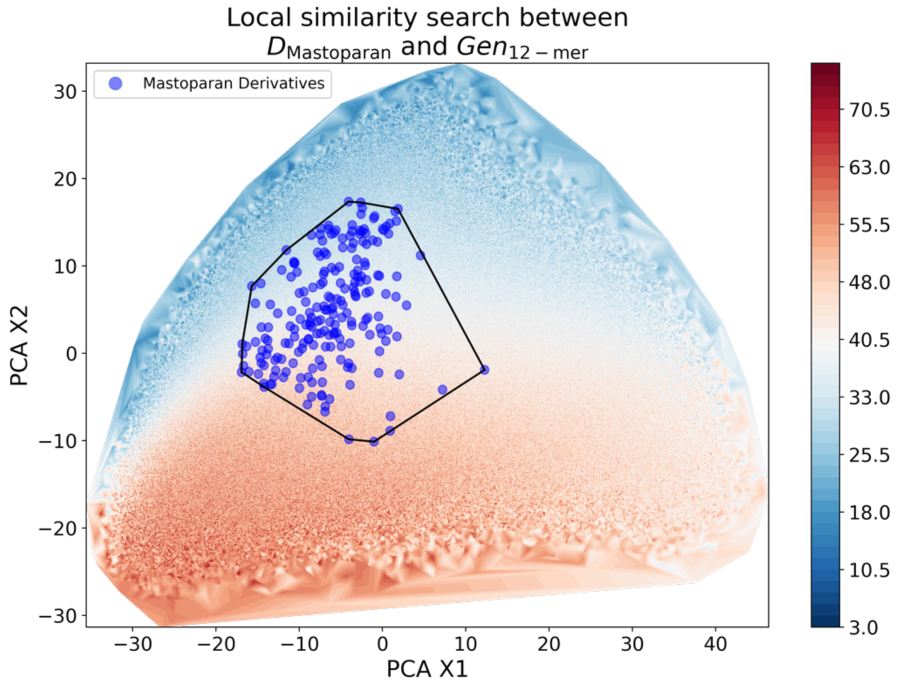

Local similarity search between
$D_{\mathrm{Mastoparan}}$ and $Gen_{12-\mathrm{mer}}$
Mastoparan Derivatives
PCA X2
PCA X1
30
20
10
0
−10
−20
−30
−30
−20
−10
0
10
20
30
40
70.5
63.0
55.5
48.0
40.5
33.0
25.5
18.0
10.5
3.0

`

C.

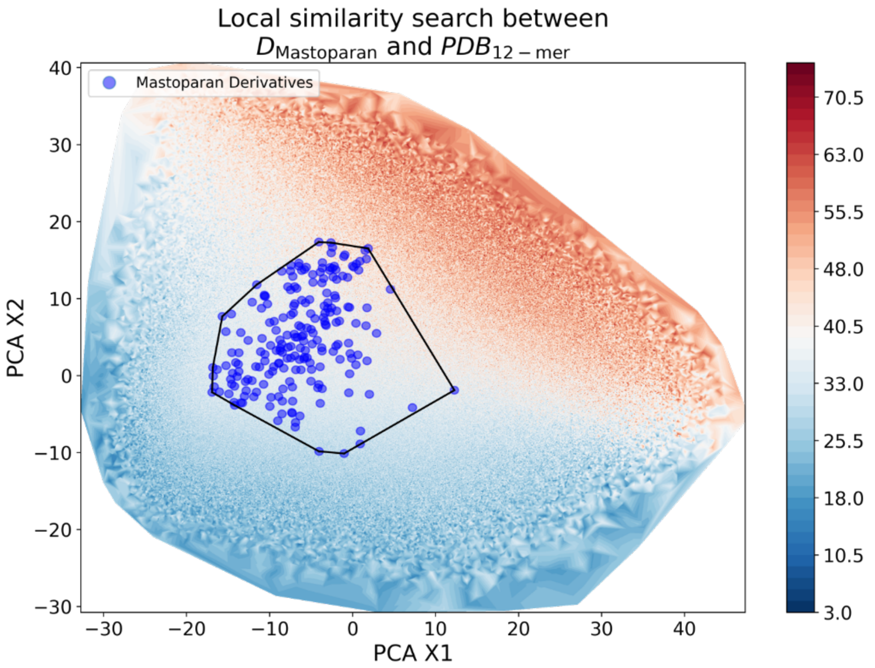

Supplementary Figure 1 | (A-B) The chemical space spanned by first 2 PCA dimensions of the latent representation of 2 randomly chosen subsets (B_{samp}) of 2M peptides each from Gen-12mer dataset. (C) Also shown is the same operation on PDB-12mer data. The black bounding box represents the convex hull of the training Mastoparan derivative peptides from $D_{mastoparan}$.

Supplementary Table 2 | Benchmarking TARSA against peptide generative models and QSAR screening models based on our Oracle proxy. For generative models (g), 50,000 peptide samples were generated using the trained model and ranked using the Oracle proxy. For QSAR screening using the Oracle proxy, 50,000 random peptide sequences were chosen from the PDB_{12mer} dataset. For Local search, 50,000 random peptides were sampled from PDB_{12mer} / GEN_{12mer} such that they are contained within a convex hull enclosing $D_{Mastoparan}$ (Appendix B). The Hit Rate (R) was calculated as $R = \frac{hits_A}{|N|} \times 100$, where $hits_A$ are the number of discovered/generated peptides with predicted % cell inhibition > 50% and |N| = 50,000. Diversity and Novelty are as defined in the metrics subsection of the methods section. Details about each baseline can be found in Appendix B. Wall Time (min) was the inference/sampling time for each method on a single Tesla V100 GPU and computing mean predicted potency with the Oracle proxy. (g): Generative Model; (g*): Screening on a peptide library constructed with generative model.
k = 100; LSTM: Long Short-Term Memory; WAE: Wasserstein Auto Encoder; PSO: Particle Swarm Optimization.

`

Baseline	Hit Rate (R) ↑	Diversity Top-K ↑	Diversity First-K ↑	Novelty Top-K ↑	Novelty First-K ↑	Wall-Time (minutes)↓	Mean Potency (predicted % cell inhibition)	
							Top-k ↑	First-k ↑
QSAR (Oracle proxy) – PDB$_{12mer}$	3.00±1.5	9.30±2.0	10.80±1.3	10.50±1.2	11.50±1.4	30.0±3.0	65.40±2.5	32.30±1.8
QSAR GEN$_{12mer}$ (g*)	3.10±1.4	7.70±1.9	8.90±1.1	10.70±1.3	11.20±1.3	35.0±2.8	65.80±2.6	37.40±2.0
Local Search PDB$_{12mer}$	1.90±1.2	8.10±1.8	8.85±1.2	10.30±1.2	11.00±1.5	62.0±3.1	45.20±2.3	35.40±1.9
Local Search GEN$_{12mer}$(g*)	1.20±1.1	7.00±1.7	7.70±1.3	9.60±1.3	10.20±1.2	60.0±3.0	46.00±2.2	30.30±1.6
Genetic Algorithm (g)	51.0±2.6	4.40±1.2	3.60±1.1	12.50±1.5	12.80±1.4	55.0±2.9	61.40±2.7	40.06±0.0
LSTM + Transfer Learning (g)	62.5±2.5	4.00±1.1	4.40±1.2	5.70±1.1	6.50±1.3	50.0±2.8	60.30±2.5	55.78±0.0
WAE + PSO (g)	70.0±2.7	5.80±1.4	6.20±1.3	8.90±1.3	9.50±1.2	65.0±3.2	58.00±2.6	56.95±0.0
PDB$_{12mer}$ *TARSA*	93±2.41	9.20±1.3	11.02±1.1	10.03±1.5	10.54±1.4	45±1.1	64.70±1.1	55.32±2.8
GEN$_{12mer}$ +*TARSA* (g*)	92±3.64	7.58±1.4	8.50±1.0	10.21±1.1	10.45±1.4	45±1.0	65.21±1.1	52.92±2.5

Supplementary Note 3: Low Dimensional State Representations

Autoencoder

In order to learn a lower dimensional representation for the peptides, a simple autoencoder comprising a bidirectional Gated Recurrent Unit[23] (GRU) (size = 80*2) encoder and an architecturally similar decoder were trained. The size of the learned representation was 50. A dropout of 0.5 was applied to each GRU and linear layer in the encoder and the decoder. A negative log-likelihood loss was used to predict the amino acid at the next timestep. Adam optimizer with a learning rate of 0.0007 was used to optimize model parameters. Early stopping was adopted to prevent the overfitting of the model. Supplementary Fig 2 shows the AutoEncoder's training and validation loss as it learns to reconstruct data. The training dataset is derived from Uniprot[24].

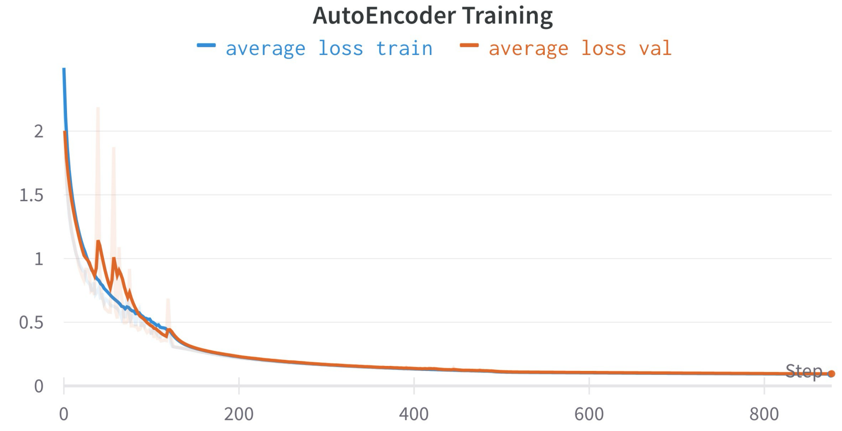

Supplementary Figure 2 | Training and Validation reconstruction loss decreases consistently with time.

`

ESM

Other amino acid sequence representations were evaluated that were pre-trained using Transformer protein language models[25]. In particular, peptide representations were retreived using the esm1 t6 43M UR50S model[26] that provides an embedding of 768 dimensions. Both the autoencoder embeddings and the ESM representations capture the high-level biochemical similarity information and hence were expected to help with the performance of downstream tasks, such as peptide classification. The representations derived from ESM model and those learned from autoencoder were used to benchmark deep representations against domain-informed descriptors. It was observed that the domain-informed descriptors outperformed deep representations and consequently the former was used to build the Oracle proxy. Moreover, our experiments established the superiority of ESM representations over AutoEncoder representations in terms of predictive performance, speed, and scalability (Fig.3A). Hence, ESM was chosen for the subsequent projection of peptides on the navigation board for policy training.

Bag-of-words with Positional Embeddings (BOWPE)

In order to experiment with extremely inexpensive low dimensional representations for the peptide sequence, bag of word (BOW) embeddings for amino acids in peptide sequences were calculated. Positional embeddings[27] were augmented to the bag of word representations to preserve the relative spatial information of amino acids within the peptide sequence.

$$X_{pep} = PE\left(g\left(S_{pep}\right)\right) + g\left(S_{pep}\right) \tag{1}$$

where, S_{pep} is the peptide sequence, g is BOW embedding, PE is position embedding defined for odd and even indices as

$$PE_{(pos,2i)} = \sin\left(\frac{pos}{10000^{\frac{2i}{d_{model}}}}\right) \tag{2}$$

$$PE_{(pos,2i+1)} = \cos\left(\frac{pos}{10000^{\frac{2i+1}{d_{model}}}}\right) \tag{3}$$

$d_{model} = 20$ was chosen, resulting in manageable representations of (12×20) for 12-mer peptide datasets, which were flattened to 240 dimensional representations before the PCA step.

From the experiments with these BOWPE representations of Gen-12mer dataset for training TARSA policy, it was observed that the modes were not well separated Supplementary Fig 3-A,B, resulting in poor convergence of the policy Supplementary Fig 3-C,D. The low mean rewards aggergated by the agent were indicative of the policy's failure. As shown in the recovered percentage plot of Supplementary Fig 3-E, the peptides investigated by the agent were almost equally likely to be of low-potency as they were to be highly active, meaning searching for high potency regions was unsuccessful. The failure of TARSA with BOWPE is largely attributed to the ineffectiveness of underlying representations in delineating high potency regions from moderate and low ones.

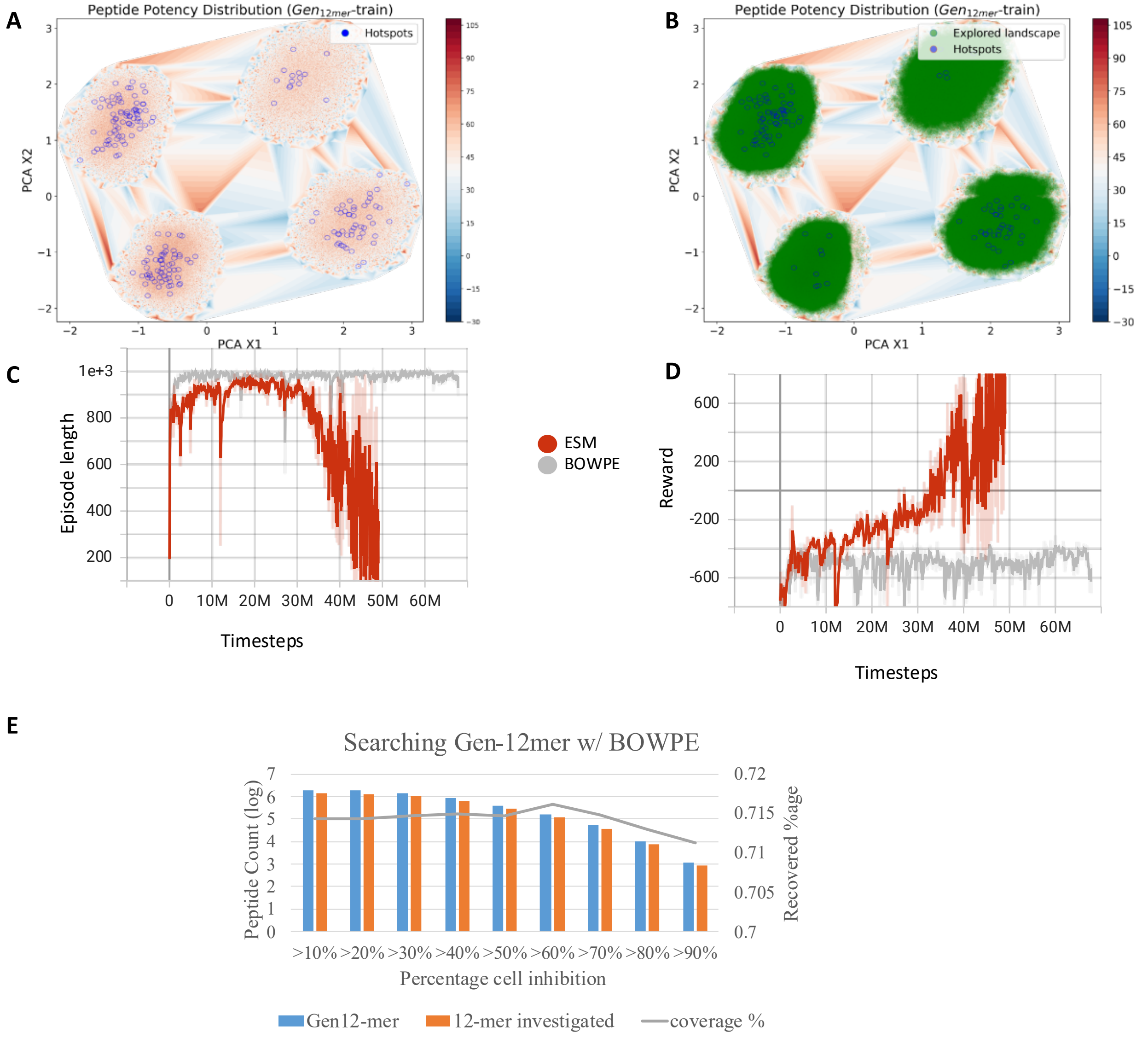

Supplementary Figure 3 | Failure modes of TARSA when used with Bag of words and positional embedding representations of peptide sequences. (A-B) Highly active peptides are clustered together with less potent peptides, indicating for representational capabilities of TARSA. (C-D) Poor convergence of TARSA with BOWPE when compared to ESM. (E) The discovered peptides show negligible enrichment.

`

Supplementary Note 4: Surrogate Functions

The higher-level architecture for all the surrogate methods described in this section remains same – leveraging MLP and CNN as feature extractor. The choice of model class was limited by the small scale of our datasets.

The modlamp descriptors for peptide sequences yield 139 dimensional representations which were reduced to a lower dimension h_{MLP}. Additionally, other peptide descriptors that have been reported to work for ACP discovery were utilized. These included AAINDEX[28] , BLOSUM62[29], one-hot encoding of amino acids, and Z-scale descriptors[30]. These were stacked to yield (L x 576) representation which too were reduced to a lower dimension h_{CNN}, where L is peptide sequence length. BatchNorm[31], average pooling, and leakyReLu were used with each layer withing the CNN module. Finally, the outcome was estimated as

$$\hat{y} = MLP_{\boldsymbol{\theta}}(\boldsymbol{h}_{MLP} \oplus \boldsymbol{h}_{CNN}) \tag{4}$$

Density weighted Mean Squared Error (MSE) for regression tasks and binary-cross entropy loss for classification tasks were used as learning objectives. In all surrogate models, an Adam optimizer with step learning rate scheduler was used. The shared hyperparameters across tasks are listed below (Supplementary Table 3)

Supplementary Table 3 | Shared architecture across surrogate models

Module	Hyperparameters	Value
CNN	Conv Output Channels	32
	Conv Kernel Size	576
	Fc1	[**256**, 128]
	Fc2	[**64**, 32]
MLP	Fc1	100
	Fc2	64
Aggregate	Fc1	128
	Fc2	64
	Fc3	32

Oracle Proxy

QSAR models rely on the correlation between the chemical structure of peptides and their biological activities, and this correlation is captured by the selected descriptors. Therefore, selecting appropriate and relevant descriptors is crucial in QSAR modeling to obtain accurate predictions of the biological activity of new peptides[32–34]. To this end, the performance of three

`

domain-informed descriptors and two data-derived representations from pretrained language models was compared. For each algorithm, Pearson correlation was chosen as the performance metric. Density-weighted MSE loss was used as the optimization objective for experiments with MLP. We employed grid search approach to tune all hyperparameters. Specific values used are listed in Supplementary Table 4. The results were 5-fold cross-validated (Supplementary Fig 4). The dependence of inductive features on commercial software MOE restricts its scalability and hence for easy dissemination and reproducibility of our work, we choose the modlamp and ifeature descriptors for subsequent development of the Oracle proxy. The true potency of bioactive peptides may be realized only following an extensive wetlab-screening. However, such protocols are prohibitively expensive to be carried out at scale. To combat this expense, peptide potency may be approximated using neural networks. An ML-based proxy to predict peptide potency was trained using the aforementioned modlamp and ifeature descriptors and a fusion architecture built upon MLP and Convolutional Neural Network.

Supplementary Table 4 | Grid search for hyperparameters. The optimal values for Oracle proxy are highlighted in **bold**. Learning rate is on 10^{-4} scale.

**Hyperparameter**	**Values**
Learning rate	[1,**2**,5]
Minibatch	[32, **64**, 128]
Dropout	[**0.3**, 0.5]
α (density weighted MSE)	[**0.5**, 0.8, 1.0]

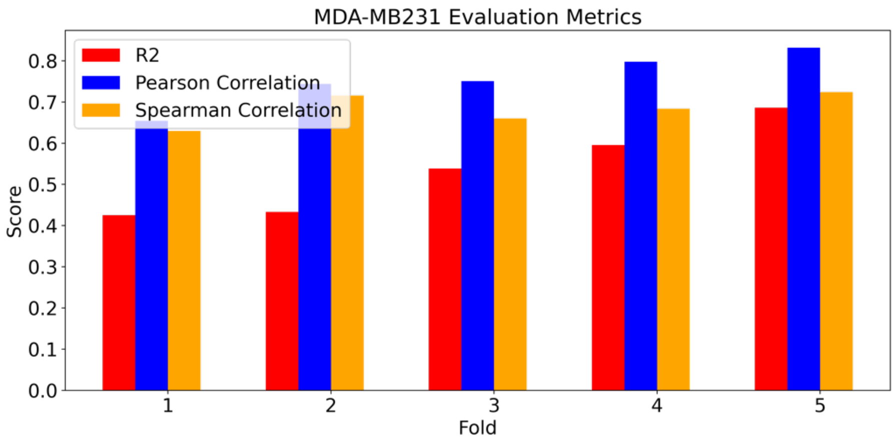

Supplementary Figure 4 | Oracle proxy performance across 5 folds for $D_{Mastoparan}$

ACP classification

`

An additional level of filtering was employed through an ACP classification model trained on a heterogeneous ACP dataset derived from the CancerPPD[35]. We considered the positive examples as the ACPs provided within CancerPPD, while negative samples were mined from UniProt database following the strategy presented in Vijayakumar et.al[36]. The modeling approach is the same as Oracle proxy with the exception of the classification head, which is a single dense layer with one output neuron and a sigmoid activation function to predict the probability of belonging to one of the two classes (ACP/non-ACP).

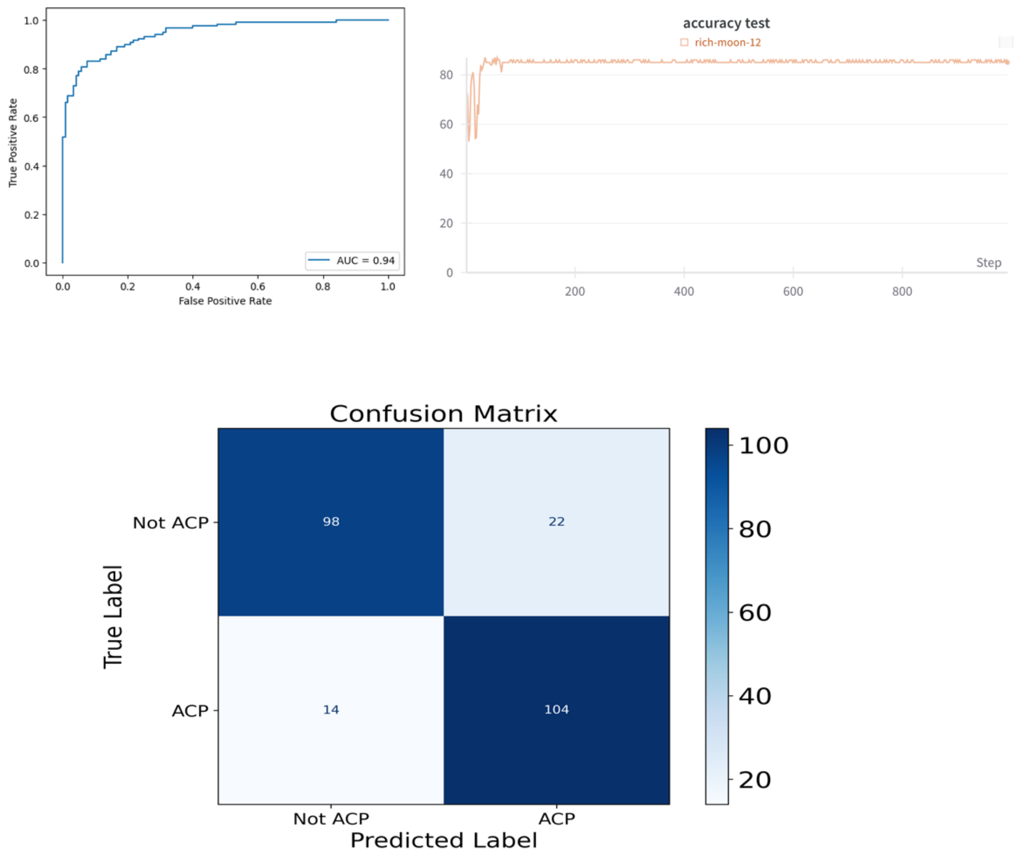

Supplementary Figure 5 | ACP classification filter trained on heterogeneous public dataset - CancerPPD

`

Cytotoxicity

The cytotoxicity filtration module was built along the same principles as the Oracle proxy. The percentage PBMC cell inhibition of singly substituted Mastoparan derivatives within the $D_{mastoparan}$ dataset was utilized as labels to train this regression model. The same hyperparameters as the Oracle proxy were found to be optimal. The model's performance across the five folds of five-fold cross validation for the toxicity prediction regression problem is depicted in Supplementary Fig 6, demonstrating consistent high Pearson and Spearman correlations.

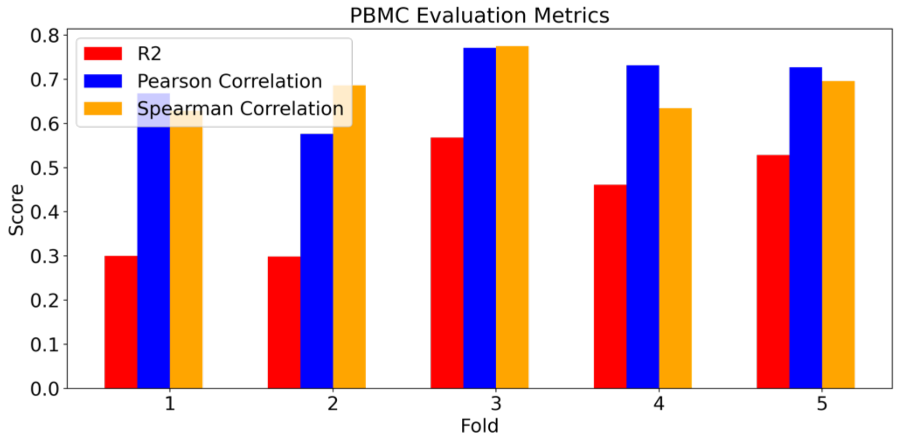

Supplementary Figure 6 | Model performance metrics (R2, Pearson Correlation, and Spearman Correlation) across five independent folds for the cytoxicity prediction regression problem.

Cytotoxicity classification

A broader cytotoxicity classification was developed using the toxinpred dataset[37] . This dataset comprises of 8233 toxic proteins and 460,257 non-toxic proteins from UniProt[38] and Swiss-Prot[39] databases built using keyword filtering and sequence length cutoffs. Here too, we used the same modeling strategy as the ACP classification model (NNTox) discussed above. We combined the main and independent sets provided by Sharma et. al.[37] for our training and validation. Supplementary Fig 7 shows that area under the curve of Receiver Operating Characteristic (ROC) curve of NNTox is 0.98, indicating its effectiveness in distinguishing toxic peptides from non-toxic ones.

`

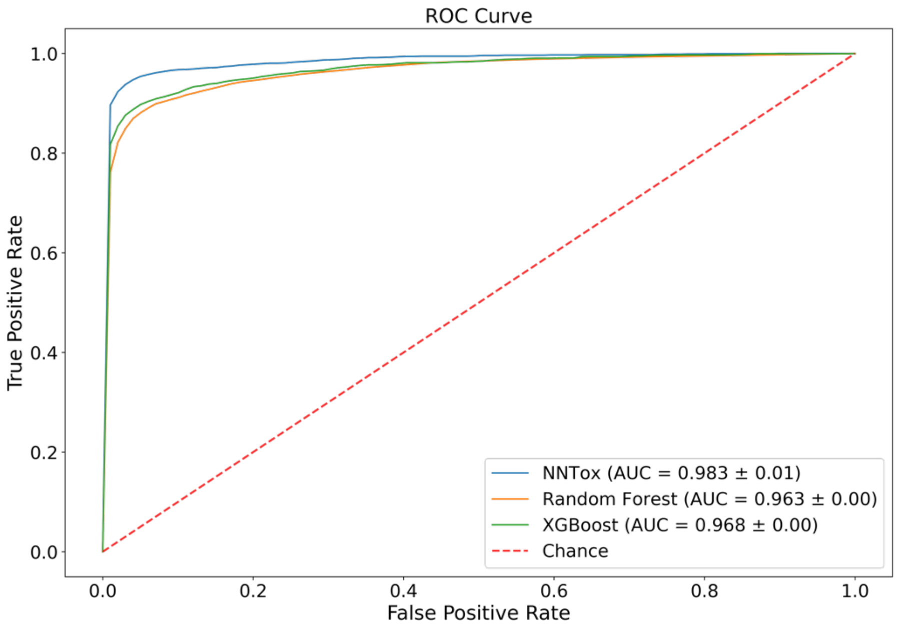

Supplementary Figure 7 | Cytotoxicity classification filter trained on public dataset – ToxinPred. The area under the curve of ROC curve of NNTox model outperforms the random forest and XGBoost baseline models.

Supplementary Note 5: TARSA

Method

Algorithm 1: Target Adaptive Reinforcement learning for Sampling Activity Landscape (TARSA)- training

Input: D_{train}: 2D-PCA of B_{train} samples from library D

$r(s, a)$: Reward function

B_{train}, B_{samp}: Extrinsic batch sizes

i: hotspot update frequency

Output: Trained policy $\pi_{\phi,\psi}$

/* Rollout Phase: Collect a trajectory of transitions $\{s_t, a_t, r_t, s_{t+1}\}$ */

p, q = min(PCA(D_{train})), max(PCA(D_{train}))

$h_{target} \sim U(p, q)$

for *episode = 1,2,....* **do**

  $X_0^a \sim U(p, q), X_0^g = h_{target}$

  $s_0 = (X_0^a, d_0^{ag}, X_0^g)$

  while $t < T$ ***and*** *not done* **do**

    $a_t \sim \pi_{\phi,\psi}(. \mid s_t)$

    $r_t, s_{t+1} = TRANSITION(s_t, a_t)$

    store transitions : $\tau \rightarrow \tau + (s_t, a_t, r_t, s_{t+1})$

    $s_t = s_{t+1}$

    if d_t^{ag}*==0* **then**

      done =True // agent reached target

    if *episode%i==0* **then**

      $X_t^g = h_{target} = UPDATE - HOTSPOT(H_c)$

      done = True

    /* Learning Phase: Update Policy (Actor) & Value (Critic) Networks */

    Optimize PPO Loss wrt ϕ, ψ

    $\phi \leftarrow \phi_{new}, \psi \leftarrow \psi_{new}$

    t++

  episode++

return $\pi_{\phi,\psi}$

Algorithm 2: TRANSITION

Input: State: s_t, action: a_t, reward function: $r(s, a)$, oracle proxy: $\mathcal{F}_p$, rotation matrix:$\mathbf{R}$

Output: Next state s_{t+1}, reward r_t, flag *done*

$X_t^a, X_t^g, d_t^{ag} \leftarrow s_t$

$\{\lambda_t, \theta_t\} \leftarrow a_t$

$X_{t+1}^a = \lambda_t \times [\mathbf{R}_{\theta_t}^T X_t^a]$

$r_t = r(s_t, a_t)$ // query reward function at location X_t^a

if $\mathcal{F}_p(X_{t+1}^a) > \mathcal{F}_p(X_t^g)$ **then**

  $X_{t+1}^g = h_{target} = X_{t+1}^a$ // update goal if more potent ($\mathcal{F}_p$) candidate found

  $H_c \rightarrow H_c + h_{target}$

  done = True

d_{t+1}^{ag}=$\|X_{t+1}^a - X_{t+1}^g\|_2$

$\mathrm{s}_{t+1} \leftarrow X_{t+1}^a, X_{t+1}^g, d_{t+1}^{ag}$

return r_t, s_{t+1}, h_{target}, done

Algorithm 3: UPDATE-HOTSPOT

Input: H_c, rollouts: τ

Output: updated h_{target}

for h_i *in* H_c **do**

  Retrieve rewards R_i for trajectories when $\mathrm{h}_{target} = h_i$ i.e. $\tau_i \sim \pi_{\phi,\psi}(. \mid .; h_i)$

  Draw posterior samples for h_i using MCMC: $\mu_i^* \sim P(\mu_i \mid R_i; h_i)$ and $\sigma_i^* \sim P(\sigma_i \mid R_i; h_i)$

  Store $X_i \sim N(\mu_i^*, \sigma_i^*)$

  $\mathrm{h}_{target}^{updated}$ is h_i corresponding to max value of X_i i.e. $\mathrm{h}_{target}^{updated} = H_c[\underset{i}{argmax}(X_1, X_2, ...)]$

  return h_{target}

`

Algorithm 4: TARSA Inference

/* TARSA inference (screening) */
Input: ϕ, ψ, eval batches: $\frac{D_{train}}{B_{samp}} - 1$, H_c, num-rollout, p: min(PCA(D_{train})), q: max(PCA(D_{train})), potency-threshold, k
Output: Output: top-k candidates
Data: 2D PCA of D_{eval}

for b =1,2,.....,$\frac{D_{train}}{B_{samp}} - 1$ **do**
  for r =1,2,.....,*num-rollout* **do**
    $X_0^g \sim H_c$ /* hotspot sampling proportional to estimated μ_i, σ_i $\forall h_i \in H_c$ */
    $X_0^a \sim U(p, q)$
    $s_0 = (X_0^a, d_0^{ag}, X_0^g)$
    while $t < T$ **do**
      $a_t \sim \pi_{\phi,\psi}(. \mid s_t)$
      $r_t, s_{t+1} = TRANSITION(s_t, a_t)$
      if $r_t > threshold$ **then**
        candidates $\leftarrow$ candidates+($[X_t^a, b]$)
      $s_t = s_{t+1}$
      $t{+}{+}$
    $r{+}{+}$
  $b{+}{+}$
return sorted(candidates)[:k]

Training

PPO algorithm was leveraged for policy training in TARSA. The architecture of the actor and critic networks were identical and consisted of 2 fully connected layers of 64 nodes each. Both the actor and critic networks take the environment state consisting of the agent's location and h_{target} as input. The actor-network learns 2 distributions over actions λ and θ, while the critic network outputs a single scalar value quantifying the estimate of the expected return that the agent can expect to receive from that state. Policy training for κ–TARSA P.S. on the PDB-12mer dataset was carried out for 20 GPU days on a single Tesla V100 GPU. However, policy training for PDB-large was carried out for much longer - 32 GPU days on 4 Tesla V100 GPUs.

`

Supplementary Table 5 | PPO hyperparameters used for TARSA training.

Hyperparameters	Value
Learning rate	$2x10^{-4}$
Minibatch size	64
Discount γ	0.99
Entropy coefficient c	0.03
Clip	0.2
GAE λ	0.95
Gradient clipping	0.5

Supplementary Note 6: TARSA Screening

On validity of policy transfer for offline inference

The parallelizability of TARSA is afforded due to the IID assumption of the peptide samples in large libraries. Here, we argue that the batches used for screening follow the same distribution as the training batch and hence a policy trained on training batch can be used for inference on other batches from the same library.

Let $\boldsymbol{\mathcal{D}}$ denote the dataset to screen, such that $\mathcal{D} = \{x \sim P(\boldsymbol{X}): \boldsymbol{X} \in \mathbb{R}^3\}$. Random batches are formed from the data as $B_i \subseteq \boldsymbol{\mathcal{D}}\ for\ i = 1,2, \dots, n$ and $\bigcup_{i=1}^{n} \boldsymbol{B}_i = \boldsymbol{\mathcal{D}}$. For $Y_k \sim \boldsymbol{B}_i, Z_k \sim \boldsymbol{B}_j, X_k \sim \boldsymbol{\mathcal{D}}\ \forall\ i,j \in \{1,2, \dots . m\}$, samples followed the same probability distribution as the underlying distribution of $\mathcal{D}$ i.e. $P(Y_k) = P(Z_k) = P(X_k)$. Moreover, since all samples were drawn from the same population without any preference, they were identically distributed.

We empirically showed that these assumptions hold by comparing batches from PDB-large dataset. Firstly, the densities of first two Eigenvectors for the training batch and a randomly chosen disjoint batch of 2M samples used for testing were compared. A small KL divergence between these densities implied that the samples in these batches had near identical spatial distribution in 2-D space spanned by these Eigenvectors. (Supplementary Fig 8). The Kullback-Leibler divergence between Kernel Density Estimates (KDE) was approximately zero (Supplementary Fig 9). Next, the 3rd dimension for sampled peptides corresponded to the predicted potency and was visually shown to be near identical (Supplementary Fig 10) . Taken together, the training and the testing batches had identical distributions and hence were suitable for screening with an offline trained policy.

`

It was also shown that the offline policy-transfer strategy may still hold for reasonably similar datasets (PDB-12mer, Gen-12mer) (Supplementary Fig 11)

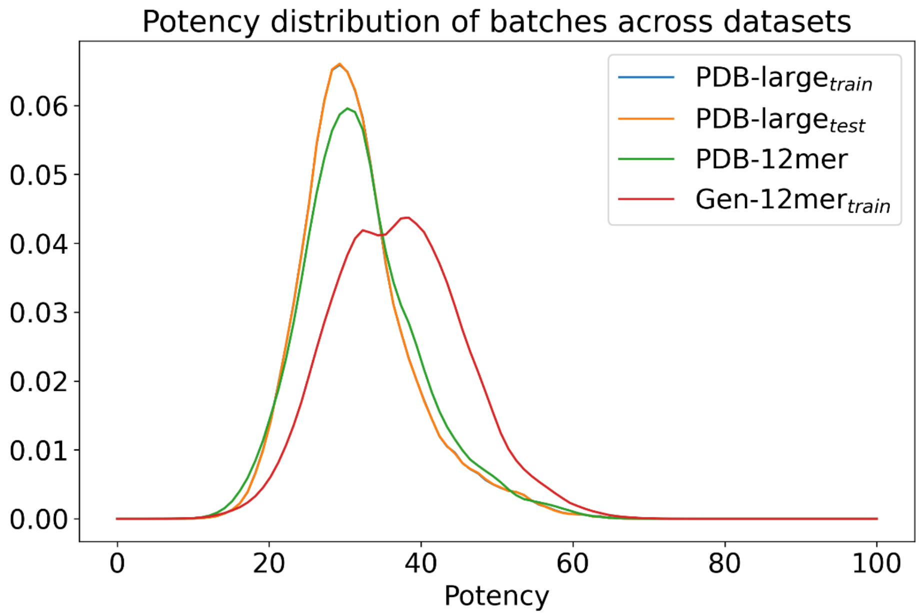

Supplementary Figure 8 | Comparing Kernel Density Estimates for predicted potency between PDB-large train batch against a randomly sampled test batch of the same size, the KL divergence is 1.6×10^{-5} i.e. almost identical distributions. PDB-12mer also has a close enough distribution with KL divergence 1.0×10^{-2}, meaning a policy trained on PDB-large$_{train}$ may still be applicable to PDB-12mer. Gen-12mer$_{train}$ has a significantly different distribution (KL-divergence 0.24) to any other dataset batches indicating a need for a fresh policy training.

`

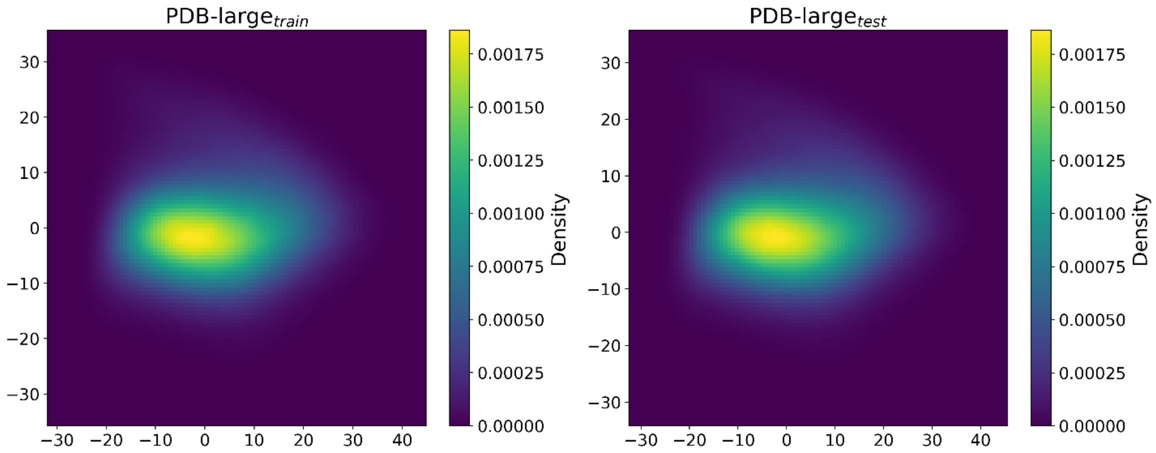

Supplementary Figure 9 | Comparing KDE for first two Eigenvectors potency between PDB-large train batch against a randomly sampled test batch of the same size, the KL divergence is $2.3x10^{-5}$ i.e. almost identical distributions.

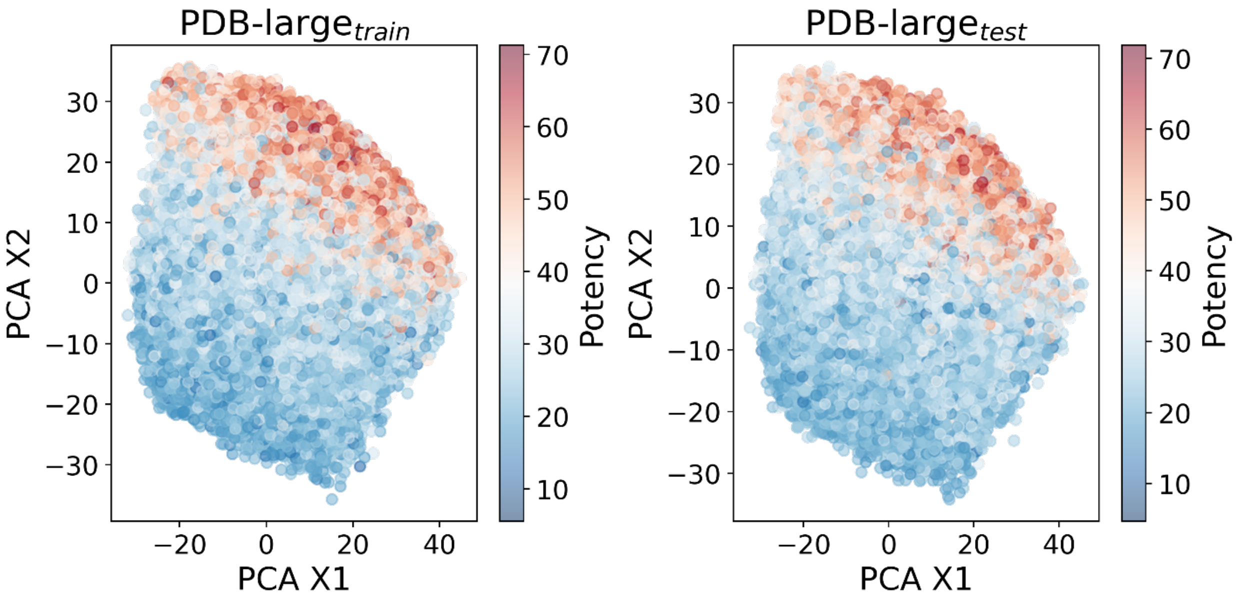

Supplementary Figure 10 | Comparison through visualization: First two Eigenvectors and predicted potency**.**

`

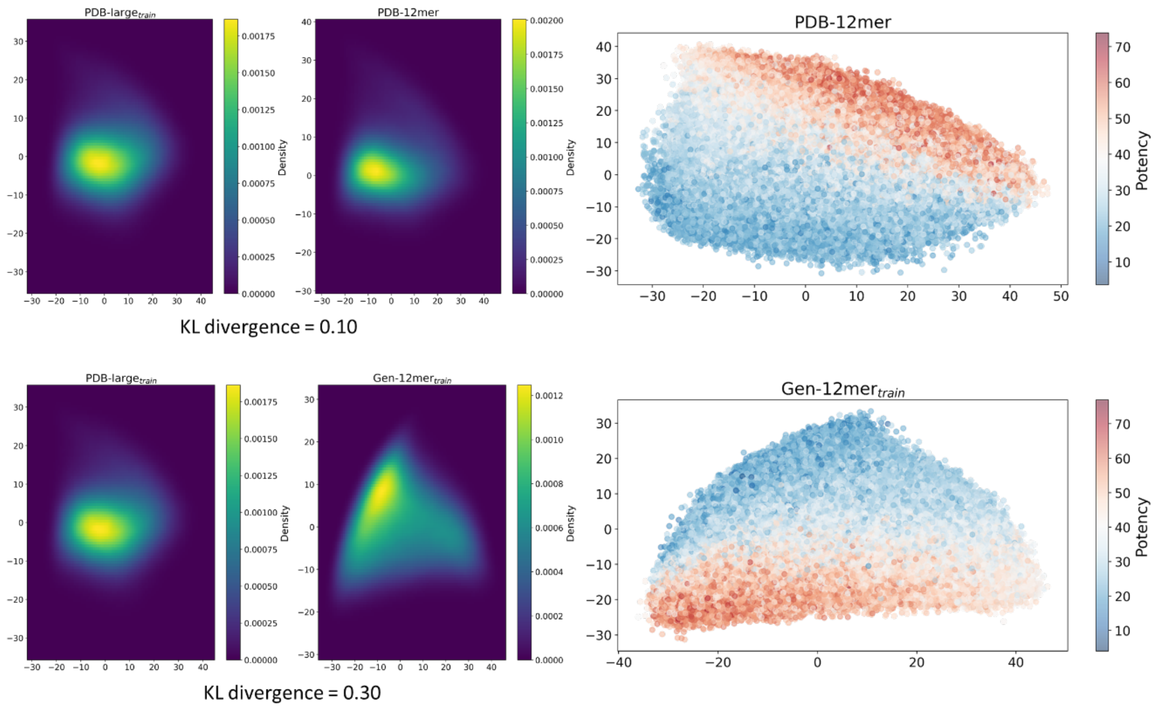

Supplementary Figure 11 | Comparing distributions of PDB-12mer and Gen-12mer to PDB-large. The top row compares distribution of PDB-12mer to PDB-large and bottom row compares to Gen-12mer to PDB-large. The left two columns indicate the distribution of peptides along the first two principal Eigen vectors obtained from PCA decomposition. The rightmost column shows the peptide potency distribution for the PDB-12mer and Gen-12mer.

TARSA inference strategies and discovery termination

In addition to the policy π, the trained κ-TARSA P.S. also learns a set of candidate h_{target} and the corresponding estimated posterior for their potency rewards H_c. In order to choose the right h_{target} during the inference (discovery) phase of the κ-TARSA P.S. algorithm, two different strategies were adopted - (i) SampleMu, and (ii) HighMu. In SampleMu, a sample from $N(\mu_i,\sigma_i)$ $\forall(\mu_i,\sigma_i) \in H_c$ was drawn. The h_{target} corresponding to the maximum sample was chosen as h_{target} for the subsequent 10,000 episodes. Samples were drawn to decide h_{target} after every 10,000 steps until one of the termination criteria was met. In HighMu, the top 70% h_{target}'s based on their posterior's μ_i were selected. Similar to SampleMu, 10,000 episodes for the chosen h_{target} were run before moving on to the next h_{target} or terminating the discovery process.

`

Termination Criteria: Two predetermined stopping conditions for the inference using the PepSce algorithm are proposed: (i) Time Bound, and (ii) Potency Bound. PepSce can be used with either of the two conditions. As the names suggest, the time-bound criterion terminates the inference if the allocated time budget for discovery is exhausted. Similarly, if the average potency of the discovered peptides reduces below a predetermined threshold for k consecutive episodes (5000 in our experiments), the inference was terminated.

Demonstrative Experiment: A comparative study to evaluate the performance of the two h_{target} selection strategies and the termination conditions under a controlled environment was performed. To this end, the trained policy π was run on a 2M peptide subset of the PDB-large dataset on 1 Tesla V100 GPU. For comparative purposes, the inference was run beyond the termination condition in these experiments (Supplementary Fig. 12). On average, the SampleMu strategy yielded more potent peptides than HighMu strategy. At the same time, to discover the same number of peptides, SampleMu was slower in comparison to HighMu. For an arbitrarily chosen potency threshold of 41.75, SampleMu yielded almost twice as many peptides as that from HighMu. However, if in time-bounded experiments (1200 minutes in this example), HighMu discovered slightly more peptides albeit of lower potency. This tradeoff meant that the choice of the strategy and termination condition depends on the desired criteria of a particular experiment but generally, SampleMu appeared to be the superior choice for the tested datasets.

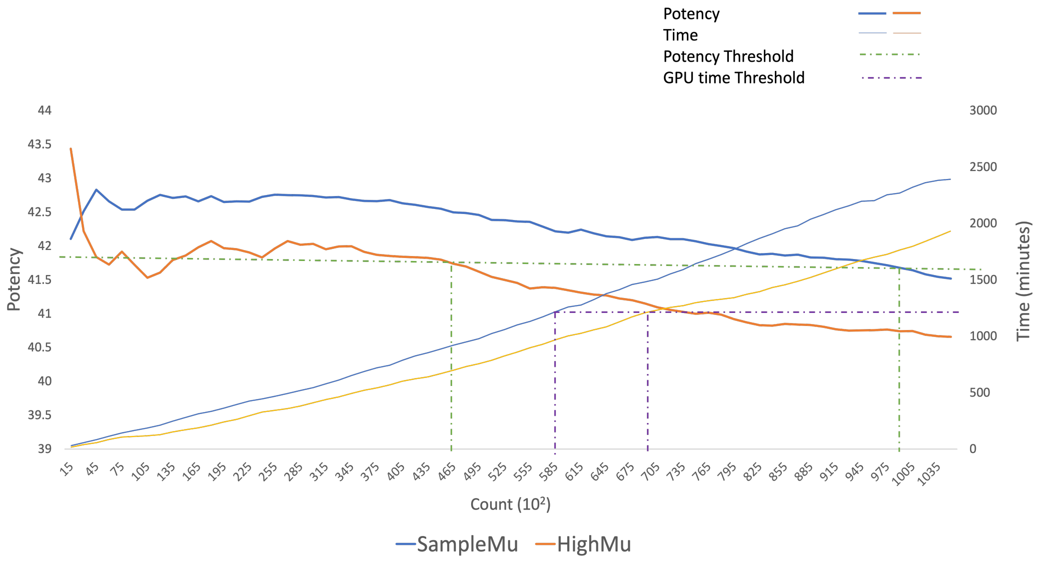

Supplementary Figure 12 | Evolution of peptide discovery with κ-TARSA P.S. algorithm and stopping condition based on time and average potency threshold.

TARSA variants

`

Supplementary Table 6 | Hotspot selection strategy by TARSA variants: f(x) is the predicted potency of an encountered peptide by the Oracle. ~ PS is the posterior sampling protocol over candidate hotspots Hc.

**Method**	$\mathbf{H_c}$	h_{target}
greedy-TARSA	-	$argmax_x f(x)$
TARSA P.S.	$\{x \mid f(x) > \max f(\boldsymbol{H_c})\}$	~ PS($\mathbf{H_c}$)
κ–TARSA P.S.	$\{x \mid f(x) > \kappa * \max f(\boldsymbol{H_c})\}$	~ PS($\mathbf{H_c}$)

Supplementary Table 7 | Performance of three PepSce variants trained in our work. All the three methods were trained for three days on 1 Tesla V100. Hotspot coverage is defined as the ratio of the area of the minimum bounding box spanned by all the peptides discovered by that method to the area of the navigation board containing all peptides in the PDB-12mer dataset. Btrain=Bsamp= 100K. Policy (π_{θ_1}) trained on PDB-12mer is considered. $\kappa = 0.9$.

Method	Hotspot Coverage	Top-100 Diversity	Top-100 Mean Potency
greedy – TARSA	0.25	8.12	60.83
TARSA P.S.	0.53	8.80	59.98
κ–TARSA P.S.	0.67	9.20	64.70

Results

In Silico Validation of TARSA discovered motifs from $\mathbf{PDB_{large}}$

Identified peptide motifs align with domain knowledge: The top 482 motifs discovered from screening PDB_{large} with $\pi_{\theta 1}$ were compared to CancerPPD, revealing leucine, lysine, and isoleucine as the most prevalent amino acids (Supplementary Fig 13). The cationicity of lysine enables interaction with the negatively charged membranes of cancer cells, causing disruption and cell death[40]. Peptides containing hydrophobic leucine and isoleucine penetrate and destabilize cell membranes, a trait common in cancer cell-killing peptides as seen in CancerPPD[41]. Conversely, low glutamic and aspartic acid prevalence was due to their negative charge, reducing membrane interaction[42,43]. Asparagine's hydrophilic nature promotes hydrogen bond formation with the aqueous solvent, thereby hindering membrane penetration. The distribution of top ACP-influencing features for these motifs lay intermediary between the $D_{Mastoparan}$ and CancerPPD

`

datasets. This indicated the model's ability to generalize beyond just the amino acids observed within the training set ($D_{Mastoparan}$) and emphasized important physiochemical properties (Fig.5D-F). For the peptide motifs discovered by running TARSA policy on the PDB_{large} dataset, a notable departure from the distribution of amino acids in known ACPs was observed for proline, glycine, and cysteine. While the CancerPPD dataset consists of peptides with all secondary structures, our modeling focuses only on alpha-helical peptides. Proline and glycine are known to be alpha-helix breakers due to their rigid cyclical structure and high conformational flexibility, respectively[44]. Hence, despite their frequent occurrence in ACPs in general, they are rare in the alpha-helical peptides discovered by our approach (Supplementary Fig 13). Although Alanine is an alpha-helix promoter, it is not a highly represented residue observed in $D_{Mastopran}$ and consequently among the discovered peptides. However, leucine, which prevails in the residue distribution, compensates for this role as the second-best promoter of alpha helix stability[45]. Beyond leucine enrichment, bulkier hydrophobic residues such as tryptophan, isoleucine, phenylalanine, and valine, along with positively charged residues like lysine, appear to dominate in representation. This suggests that these residues possibly provide additional stabilizing interactions or functional advantages in the context of membranolytic alpha-helical ACPs. Furthermore, the relative homogeneity of the Mastoparan dataset is limiting, further exploration by utilizing the hits discovered in this study will be beneficial.

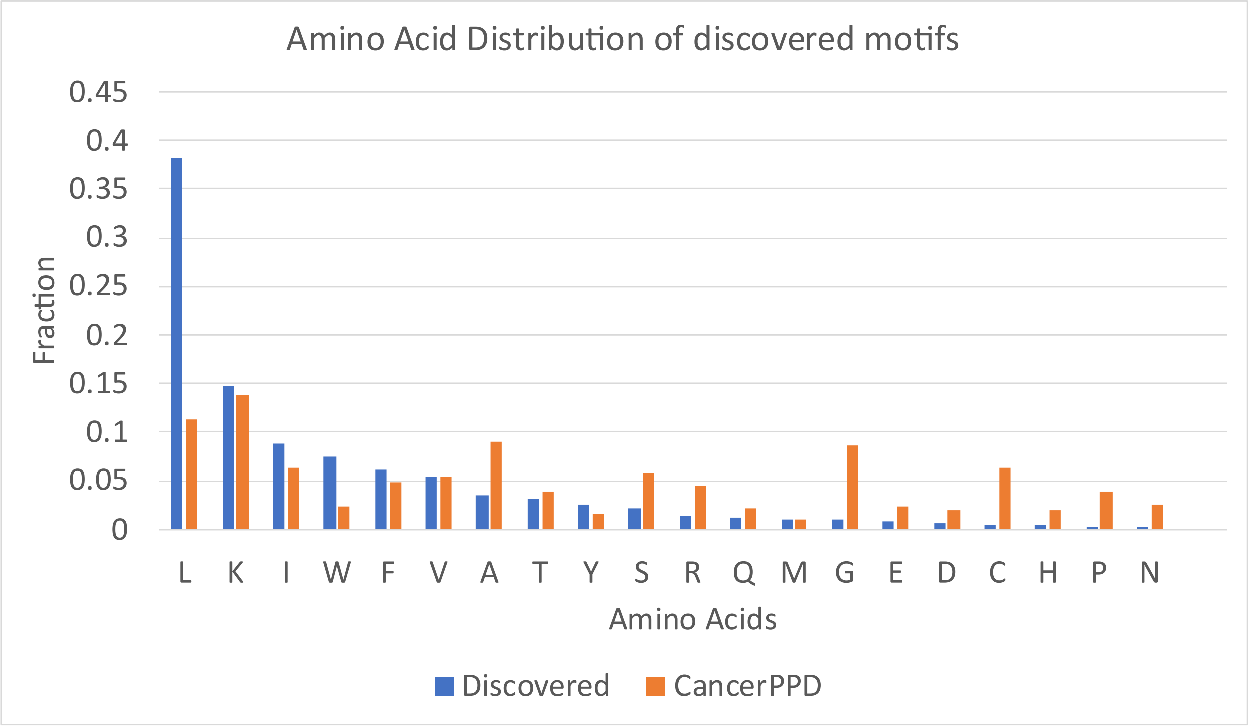

Supplementary Figure 13 | Relative frequency of 20 natural amino acids in the top discovered peptide motifs screened from PDB-large dataset. Hydrophobic and cationic amino acids are the most prevalent.

`

Resonance between discovered motifs and known ACPs: Insights from PDB metadata: Manual inspection of PDB entries linked to motifs discovered by TARSA indicated resonance with certain biological characteristics of known ACPs. For instance, the motif "YLLKALFKAL" in the PDE3A-SLFN12 complex (PDB: 7eg0) induces apoptosis[46] in cancer cells. The "WIVIIAKYLAQWY" motif, discovered in the NEDD8-activating enzyme complex (PDB: 3gzn), is associated with MLN4924, a selective inhibitor of this enzyme essential for cancer cell growth[47]. Other strong signals came from proteins derived from organisms known to be rich sources of ACPs. "*LFYKLLI*" from maize[48] (PDB 1blf, 5ou5, 6c7n), "*LIWKLL*" from cattle lactoferrins[49] (PDB: 1b1x,1qjm,2n9y etc.), "*LLKWIKTL*" from bovine lactoferrin[50] (PDB: 6xby), "LLKWIKTL" from solitary wasp[51] (PDB: 2mjr), were also reported to form amphipathic helices, a shared characteristic of many ACPs. Several other discovered motifs originated from proteins reported to have antimicrobial bioactivity (e.g. "*LLKLLKLL*" (PDB: 7nef); "*LWKALALKL*" (PDB: 2l9a)). This is to be expected as many antimicrobial peptides have similar peptide sequence characteristics and many peptides have the ability to exert a wide range of activities, including antibacterial, antiviral and anticancer properties, to name a few[11].

Influence of Training Data on ACP Property Distributions: The CancerPPD dataset comprises a diverse collection of ACPs associated with various cancer types and secondary structures. While this diversity makes CancerPPD a valuable resource for training classification models that distinguish general ACPs from non-ACPs, it is less suited for regression-based models aimed at discovering ACPs specific to a particular cancer type, such as breast cancer. Classification models benefit from broad training data to identify generalizable features, whereas regression models require a more focused dataset to capture quantitative structure-activity relationships (QSARs) relevant to a specific biological context.

To address this limitation, the study leveraged a Mastoparan-derived in-house dataset consisting of systematically designed single-residue variants of Mastoparan, each with experimentally measured cytotoxicity against MDA-MB-231 breast cancer cells. This dataset provided a QSAR framework that enabled the model to learn fine-grained physicochemical patterns relevant to breast cancer ACPs. However, a key limitation of this dataset was its lower diversity, as all peptides shared a common helical scaffold and differed from Mastoparan by only a single amino acid substitution. Consequently, the discovered peptides exhibited physicochemical properties more aligned with Mastoparan-derived sequences than with the broader CancerPPD dataset.

As illustrated in Figure 5D-F, the key ACP properties identified through literature review and feature-ranking analysis displayed a distinct distribution in the top k = 480 peptide candidates predicted by the regression-based screening model. Since the training data primarily consisted of Mastoparan analogues, the resulting peptides retained physicochemical characteristics associated with amphipathic α-helical ACPs. In contrast, CancerPPD contains ACPs targeting a wide range of cell types and includes peptides with diverse secondary structures, such as β-sheets and disordered regions. Despite these differences, the discovered peptides still fell within the broader range of ACP properties observed in CancerPPD, demonstrating that the approach successfully identified biologically relevant ACPs while prioritizing physicochemical features specific to Mastoparan-like sequences.

`

The relatively lower variance in charge density and hydrophobic moment observed in the discovered peptides was a direct consequence of the homogeneity of the Mastoparan-derived training dataset. Since Mastoparan is a cationic, amphipathic α-helical peptide, the charge and hydrophobic moment distributions were inherently constrained. As a result, the regression model favored physicochemical properties within this narrower range, leading to a more conservative selection of ACPs compared to the broader CancerPPD dataset. This highlights an inherent exploration-exploitation tradeoff in ACP discovery. CancerPPD, with its high diversity, enables greater exploration of potential ACPs but may generalize too broadly, limiting its ability to identify ACPs with specificity for breast cancer cells. In contrast, the Mastoparan dataset facilitates more targeted exploitation, identifying peptides with high efficacy against MDA-MB-231 cells but at the cost of a more constrained physicochemical profile.

This tradeoff underscores a fundamental challenge in ACP discovery: the scarcity of high-quality, diverse datasets for training predictive models. Expanding peptide datasets with well-defined experimental measurements across multiple cancer cell types and structural classes would enhance the generalizability of computational peptide design models. Future efforts should focus on increasing dataset diversity while maintaining the advantages of targeted QSAR modeling. The TARSA scheme developed in this study may offer a promising avenue for such dataset expansion, improving predictive accuracy and broadening the applicability of machine learning-driven ACP discovery.

Identified peptides penetrated cell membranes in molecular dynamics simulation: Using a coarse-grained molecular-dynamics (CG-MD) approach, interactions between the 482 discovered motifs (subsection In silico validation of discovered anticancer peptide motifs) and a lipid bilayer environment were simulated. This compared the membrane-peptide contact densities of discovered motifs and experimentally validated peptides from $D_{Mastoparan}$. The motifs' maximal contact distributions resembled those of potent breast cancer inhibitors (>40% cell inhibition). Motifs with higher membrane contacts penetrated deeper into the membrane (Supplementary Fig 18A). Peptides with <40% inhibition showed significantly fewer contacts. CG-MD confirmed that discovered ACP motifs interact favorably with cell membranes, similar to potent cancer inhibitors, indicating TARSA's effectiveness in identifying viable ACP motifs and its suitability for large-scale screening campaigns.

Sequence and Positional Analysis of Discovered Motifs: To assess the novelty of the motifs identified from PDB_{large}, their Levenshtein edit distances were computed relative to the 210 Mastoparan derivatives in the training dataset. The minimum edit distance between any discovered motif and its closest match in the training set was found to be 5, confirming that none of the motifs were exact matches to those present in the training data (Supplementary Fig 14). This statistical evaluation supports the distinctiveness of the motifs identified in PDB_{large}, reinforcing their potential as novel ACP candidates. Regarding motif prevalence, the only potent motif associated with cell inhibition exceeding 45% and found in at least three training peptides was 'LAKL'. This motif, which met the minimum criterion of a 4-mer, appeared in 150 out of the 210 Mastoparan derivatives. Positional analysis revealed that 'LAKL' consistently occurred at a fixed position, beginning at index 2, across the training dataset. This observation is likely a consequence of the

`

dataset's construction, which involved single amino acid substitutions within the Mastoparan sequence. The presence of 'LAKL' did not correlate with an increase in ACP potency. The potency distribution of peptides containing the motif mirrored the overall potency distribution of the entire training dataset, as shown in Supplementary Fig 15.

These findings suggest that while certain motifs, such as 'LAKL', are recurrent in the training data and exhibit positional constraints, their presence alone does not drive enhanced ACP potency. This highlights the importance of ML in peptide discovery, as it enables the identification of complex, non-obvious relationships that manual sequence-based analysis may overlook.

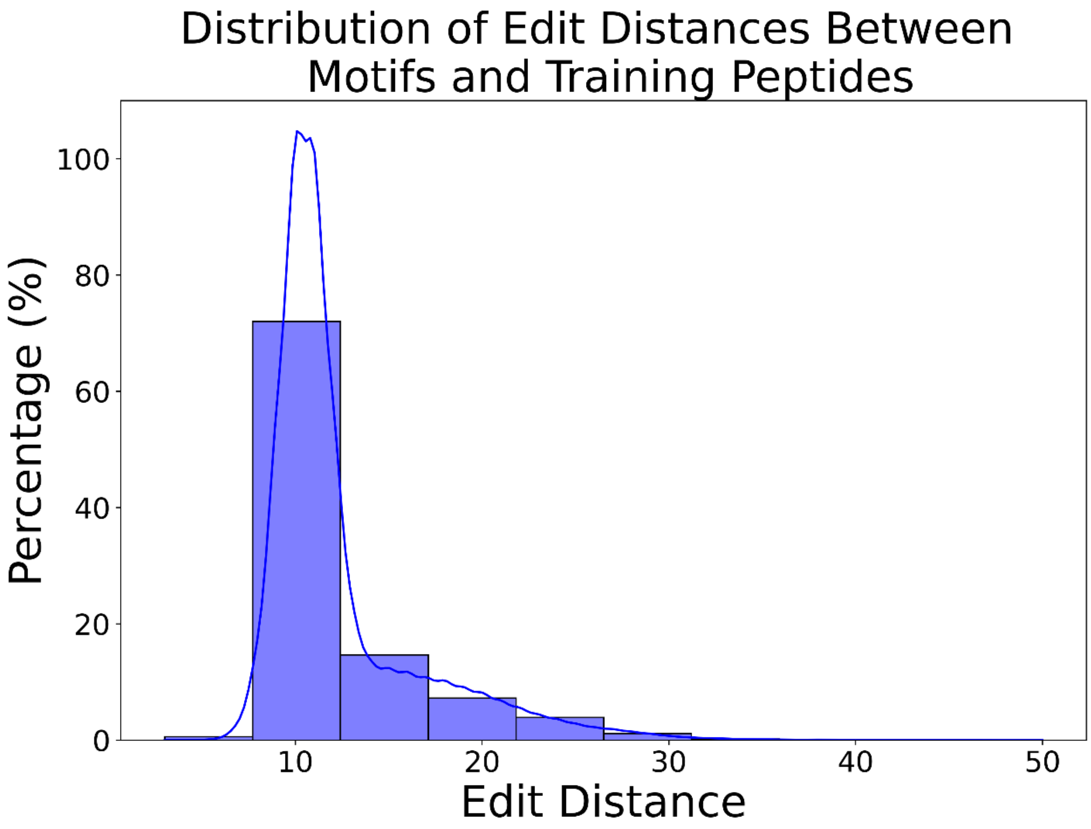

Supplementary Figure 14 | The analysis of Levenshtein edit distances confirmed that no discovered motifs from PDB_{large} were exact matches to peptides in the training set.

`

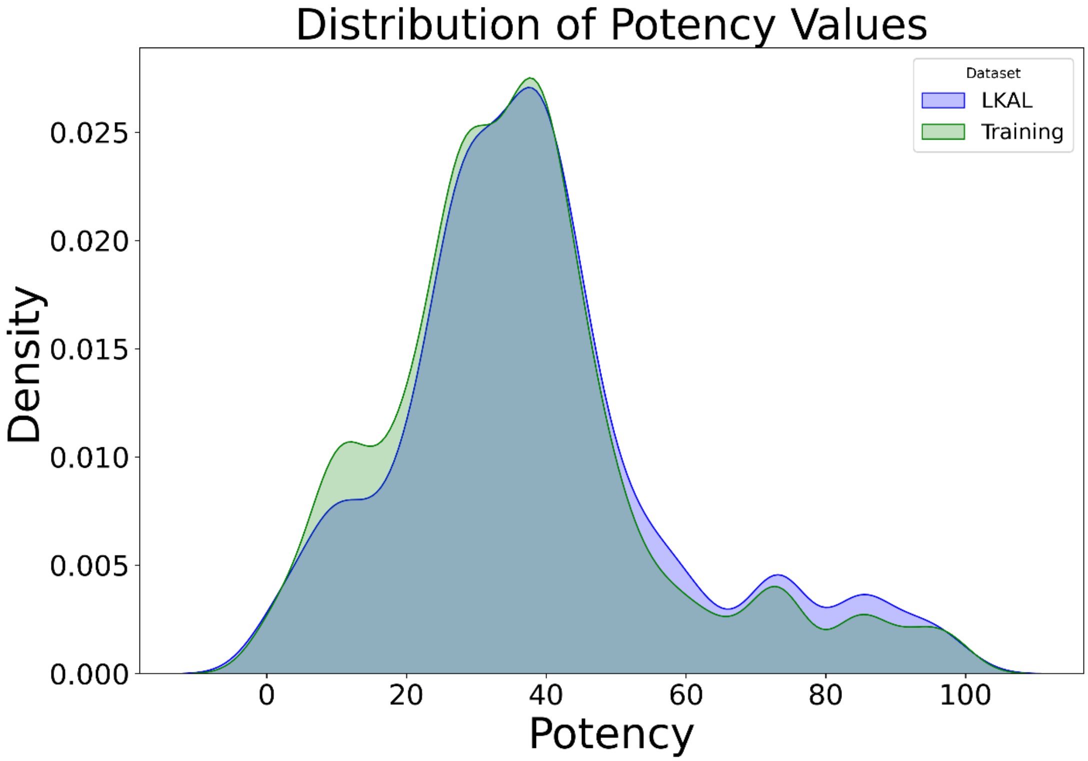

Supplementary Figure 15 | Comparison of the potency distribution of peptides containing the ‘LAKL’ motif (blue) with the overall potency distribution of the training dataset (green). The similarity in distributions indicates that the presence of the ‘LAKL’ motif does not selectively enhance anticancer potency.

`

PDB-12mer Screening

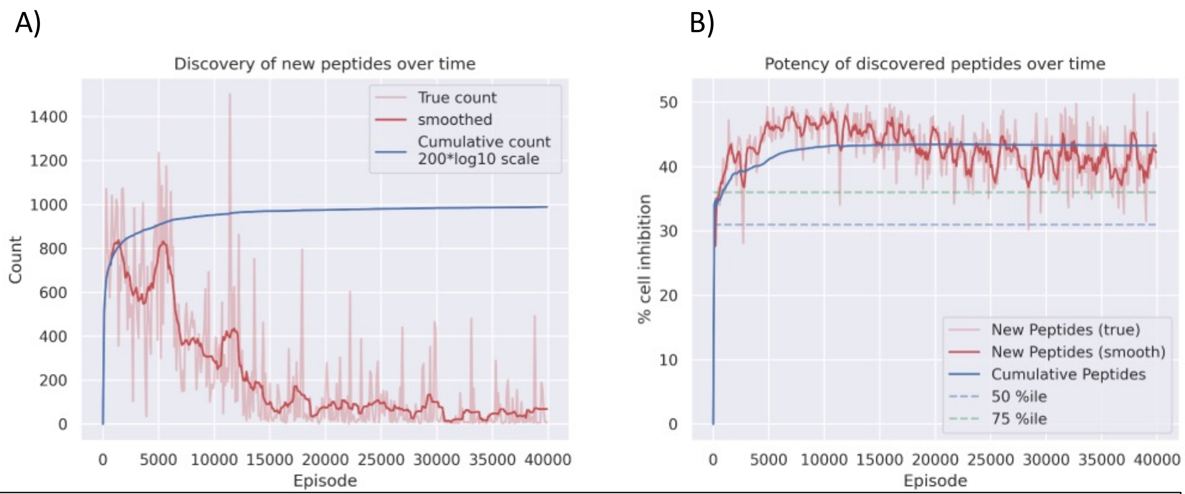

Supplementary Figure 16 | Evoluation of policy to discover potent ACP in PDB-12mer dataset. (A) As the policy evolves, the agent quickly converges to high-potency neighborhoods on peptide chemical space thereby requiring fewer peptide queries to the Oracle proxy per episode. (B): The average potency of the novel and unique peptides investigated by the agent rapidly exceeds the 75th percentile potency of the underlying dataset.

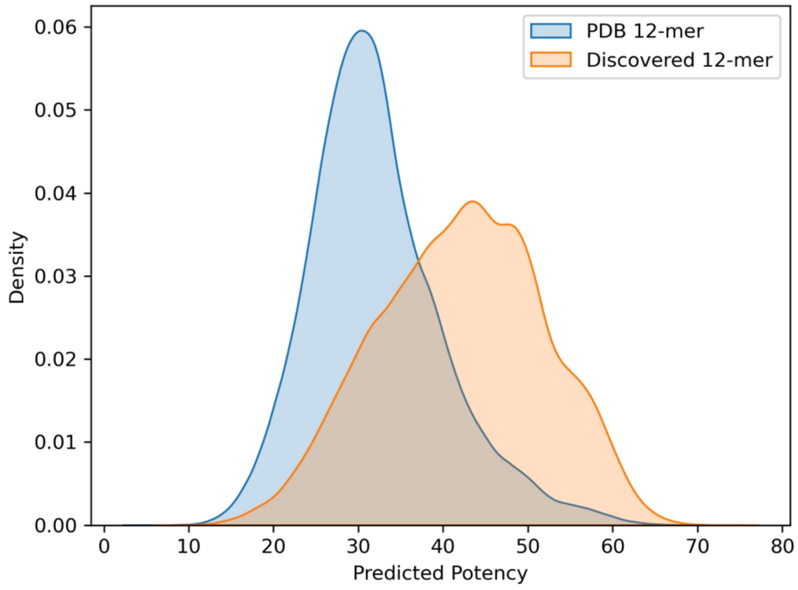

Supplementary Figure 17 | Evaluation of policy trained using κ−TARSA P.S. to discover potent ACP in PDB-12mer dataset. (A): The distribution of potency of the discovered peptides is significantly right-shifted compared to the entire PDB-12mer dataset, indicating that on average κ−TARSA P.S. has learned to locate peptides with higher toxicity towards cancer cells.

`

Supplementary Note 7: Molecular Dynamics Experiments

Coarse-grained Molecular Dynamics

Previous research suggests that the interaction of anticancer peptide ACPs with cell membranes is crucial for their cytotoxic effects[8,52]. ACP-mediated cytotoxicity is thought to occur through both membrane permeabilization and non-membrane permeabilization mechanisms, while their selectivity for cancerous cells, attributed to the higher presence of negatively charged species like phosphatidylserine (PS), is expected to reduce side effects[53]. In order to probe the interaction patterns of the 482 candidate peptides generated from the TARSA model (PDB_{large}) with lipid membranes on a large scale and under reduced computational cost, coarse-grained (CG) molecular dynamics (MD) simulations were performed[53–55]. To accomplish this, we used MARTINI 2.2 forcefield[56] and prepared peptides in α-helical form using PeptideBuilder with a constrained geometry (Φ = -60 and Ψ = -40)[57], minimized via PrepWizard[58] at a default pH of 7.0 after capping the termini. Consequently, the peptides were randomly placed (3.5nm shift) near a mixed charge-neutral 1-palmitoyl-2-oleoyl-glycero-3-phosphocholine (POPC) and negatively charged 2-Oleoyl-1-palmitoyl-sn-glycero-3-phospho-L-serine (POPS)[53,59] POPC:POPS (1:1) bilayer, which was built by *insane* tool[60], and solvated in a 90:10 ratio of TIP3P water model and antifreeze particles 'WF' within a 15 x 15 x 35 rectangular box with an optimal periodic boundary condition (with a 7nm between periodic boundary images) The system allowed it to equilibrate, then collected 100 ns of production data in three independent replicas as follows: energy minimization (steepest descent and conjugate gradient) was followed by 250 ps of NVT and 500 ps of NPT equilibration under position restraints at 323 K using V-rescale thermostat and in 15 M NaCl[8,19,52]. The peptide along with the membrane was coupled separately from the solvent and ions. Pressure coupling followed the Parrinello–Rahman scheme with semi-isotropic coupling at 1 bar. Additionally, a 1.2 nm real-space cutoff for both electrostatics and van der Waals interactions, consistent with the Martini 2.2 force field recommendations. The Verlet cutoff scheme was used with a verlet-buffer-tolerance of 0.005. During initial equilibration (NVT and early NPT stages), positional restraints were applied to both the peptide and lipid heavy atoms. Afterward, restraints were removed, and production runs ensued. All CG simulations were carried out with GROMACS 2021[61].

For analysis of MD trajectories, the MDTraj Python package was used[62]. Trajectory centroid clustering was carried with 1Visual Molecular Dynamics (VMD) using in-house TCL scripts[63]. A contact was defined whenever any bead of the peptide fell within 7.5 Å of any lipid bead (POPC and POPS)[52,59]. The contact number was computed frame-by-frame (sampled every few picoseconds), then aggregated over the three replicas to yield an average contact metric. We also computed peptide insertion depth by measuring the z-coordinate difference between the peptide's center of mass and that of the bilayer, while the backbone RMSD was monitored as an indicator of structural stability. The average backbone RMSD values across the three replicas were 35.69 ± 5.65 Å, 36.02 ± 4.87 Å, and 35.99 ± 5.16 Å for replicas 1, 2, and 3, respectively, demonstrating consistent stability among the simulations. The DRAMP database[64] provided a set of inactive peptides—with no antibacterial or cytotoxic activities—serving as benchmark references for minimal bilayer interaction. In f Supplementary Fig 19A-D, these inactive DRAMP peptides

`

appear at the low end of both contact ratio and penetration depth, thus illustrating a baseline for comparison.
Supplementary Fig 18A shows a scatter plot of the replica-aggregated contact ratio and insertion depth for four peptide categories, including DRAMP inactives, the in-house experimentally determined (< 50% or ≥ 50% inhibition) peptides, and our new generated ACP candidates. Representative last-frame snapshots (Supplementary Fig 18B) highlight the degree of membrane penetration, with some peptides (particularly the cationic and hydrophobic ones) achieving deeper insertion than the DRAMP controls. Strikingly, regardless of the category to which the peptides belong to, a mixture of membrane-ruffling, penetration, and detachment was observed. Peptides "KWLFLILSLAKYY" (generated ACP candidate), "INLKWLAALAKKIL" and "IWLKALAALAKKIL" (experimentally validated >50% BC inhibition) and even "KAAKAAKKAAKAAWK" (DRAMP inactive control) showed signs of membrane penetration. On the ither hand, peptides "YFLWNLLLLWPRV" (generated ACP candidate) and "INLKALAALAKKIQ" (experimentally validated <50% BC inhibition) displayed membrane disruption while peptides "GVITDALKGAAKTVAAELPRKAH" (DRAMP inactive control) and "INLKALAALAKKIP" (experimentally validated <50% BC inhibition) did not show visible signs of membrane penetration or disruption. Visualizations were carried out using VMD and plots were generated using VMD and in-house Python scripts[63].

In Supplementary Fig 19A–D, kernel density estimates (KDE), boxplots, and histograms further depict the distribution of contact frequencies and insertion depths. In the KDE plot, peaks highlight the most frequently observed membrane-contact extent for each peptide group. In contrast, the boxplots and histogram capture the distribution of peptide membrane-contact and insertion depths where higher contact ratio is deemed to correlate with deeper and more sustained peptide–membrane interactions—consistent with the expected disruptive behavior of potent ACP[19,65]. A trend can be observed where the generated candidate ACP peptides as well as the experimental validated >50% BC inhibitory peptides consistently display the highest contact ratio and penetration depth to the membrane center-of-mass) COM in contrast to the DRAMP inactive controls and the experimental validated <50% BC inhibitory peptides. Notably, the generated ACP peptides cover a large span of low to high membrane penetration depth, indicating high variability in perceivable ACP activity (Supplementary Fig 19C). Finally, to justify the simulation length of 100 ns, we monitored the RMSD behavior and found that equilibrium was reached within approximately 50 ns and maintained thereafter (Supplementary Fig 19E-F). In addition, the hexbin density plot (Supplementary Fig 19F) reveals that most RMSD measurements are concentrated in the central region of the heatmap, indicating that the majority of peptides converge to a stable conformation over time.

`

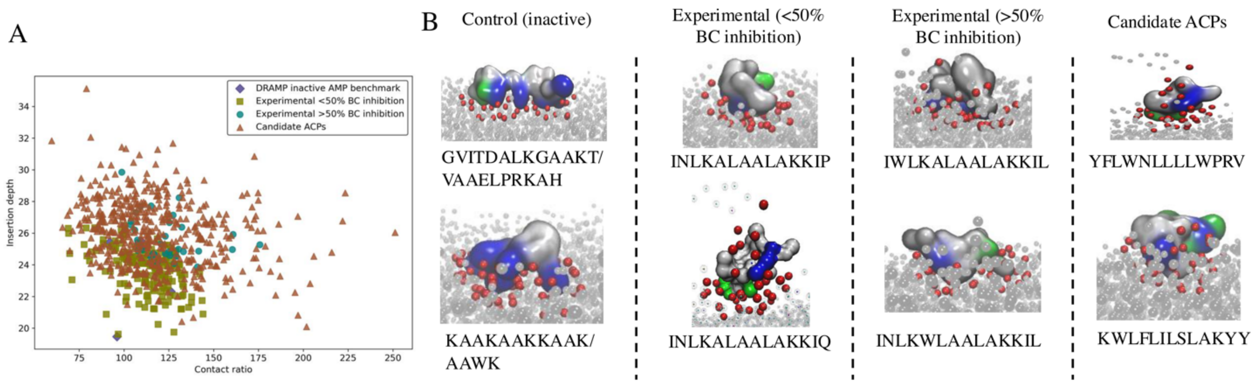

Supplementary Figure 18 | (A) Scatter plot comparing contact ratio (replica aggregated) and insertion depth (also replica aggregated) for each category (indicated in the legend box). Each point represents one peptide. Marker shape and color distinguish the four categories, illustrating any correlation between contact frequency and bilayer penetration. (B) Rendered last frame snapshots from MD trajectories of top representative examples from the following categories: literature (DRAMP database) validated inactive antimicrobial peptides (AMP) (control), experimentally determined peptides with less than 50% inhibition, experimentally determined peptides with higher than 50% inhibition, and candidate ACPs. Membrane CG residue beads within 7.5Å of peptide are depicted in red, others in grey. Polar peptide residues are depicted in green, positive-charged residues in blue, and non-polar residues in white. For long peptide sequences, the sequence is broken into two parts to fit the text box with a "/" continuator.

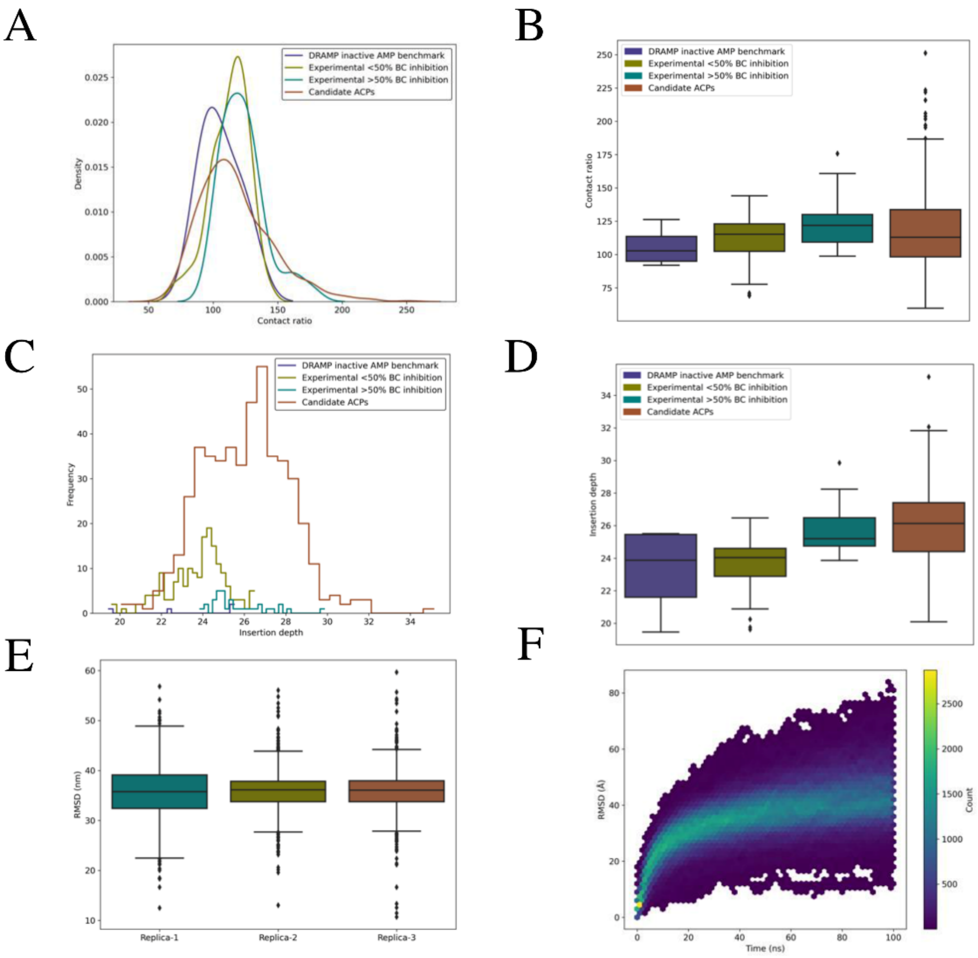

Supplementary Figure 19 | (A) Kernel density estimates (KDE) of the aggregated replica for the membrane-contact ratio for each category labeled in the legend box. Curves represent the probability distribution of contact ratio values. Peaks highlight the most frequently observed membrane-contact extent for each peptide group. (B) Boxplot of membrane-contact ratio (replica aggregated). (C) Histogram of peptide insertion depths (replica aggregated. The frequency of insertion-depth values is plotted in 30 bins. Overlaid step histograms help visualize differences in insertion profiles across categories. (D) Boxplot comparing insertion depths (replica aggregated). (E) RMSD replicate boxplot for all peptides. Boxplots of backbone RMSD (nm) for three simulation replicates. (F) Hexbin density plot of RMSD vs. simulation time (ns) for all peptides. Each hexagon's color indicates the local density of RMSD measurements over time. The color bar on the right shows the count scale. This plot reveals overall distribution and clustering of RMSD values throughout the trajectory.

All-atom Molecular Dynamics

All-atom molecular dynamics (AA-MD) simulations provide a more detailed level of molecular representation compared to coarse-grained (CG) simulations, offering insights into the specific interactions between atoms within biological systems. Here, the focus was on the AA-MD

`

simulations of the three peptides (IILKKLLDFILK, TLLTAIVKLFLK, LLQWLLKRLKAK), which were identified in our study as having the highest anticancer potency in experimental screenings. Each peptide was prepared using PeptideBuilder to generate a specific three-dimensional structure, followed by the utilization of PrepWizard[58] for further refinement and minimization, replicating the procedure used in the CG simulations.

The bilayer model for these simulations was constructed using CHARMM-GUI[66], with specific leaflet compositions of POPC:POPE:POPS:SM at a ratio of 3:2:1:1. This composition, which adds PS and sphingomyelin (SM) to the CG bilipid composition (described earlier), was chosen as a more realistic representation of breast cancer cell line membranes[67]. Consequently, the bilayer was enclosed in a simulation box with dimensions of 100 Å on each side. Following this, we used a TK console script in VMD to position each peptide over the center of mass of the membrane with a specified initial separation of 5 Å. Subsequently, PrepWizard facilitated the energy minimization of each peptide–membrane complex. The system setups were finalized using the Desmond System Setup tool, where a rectangular orthorhombic boundary box of 100 × 100 × 100 Å was defined and filled with T3P solvent to mimic physiological conditions.

Simulations were initially conducted for 500 ns and then continued for an additional 500 ns, reaching a total of 1 µs per system, following a standard Desmond protocol. The integration time step was set to 2 fs for bonded and near nonbonded interactions, maintaining the NPT ensemble at 300 K and 1 atm. For post-simulation analysis, a Python-based workflow was employed. MDAnalysis was utilized to calculate the COM distances between peptide and membrane by tracking the positions of their respective centers of mass over the full trajectory. Similarly, the backbone RMSD was evaluated against the first frame, allowing assessment of conformational fluctuations during the simulation. The first 50 ns of each trajectory were discarded in plots, and data were smoothed using an exponential moving average with α=0.005. The final averaged curves (over three independent replicas) were then plotted to elucidate peptide–membrane interactions and structural stability over the full 1 µs simulations. Representative snapshots at the start and end of each trajectory underscore the extent of membrane penetration. Interestingly, peptide TLLTAIVKLFLK did not maintain a helical structure at the end of the simulation (Supplementary Fig 20). All-atom MD visualizations were carried out using PyMol 3.1.1[68].

`

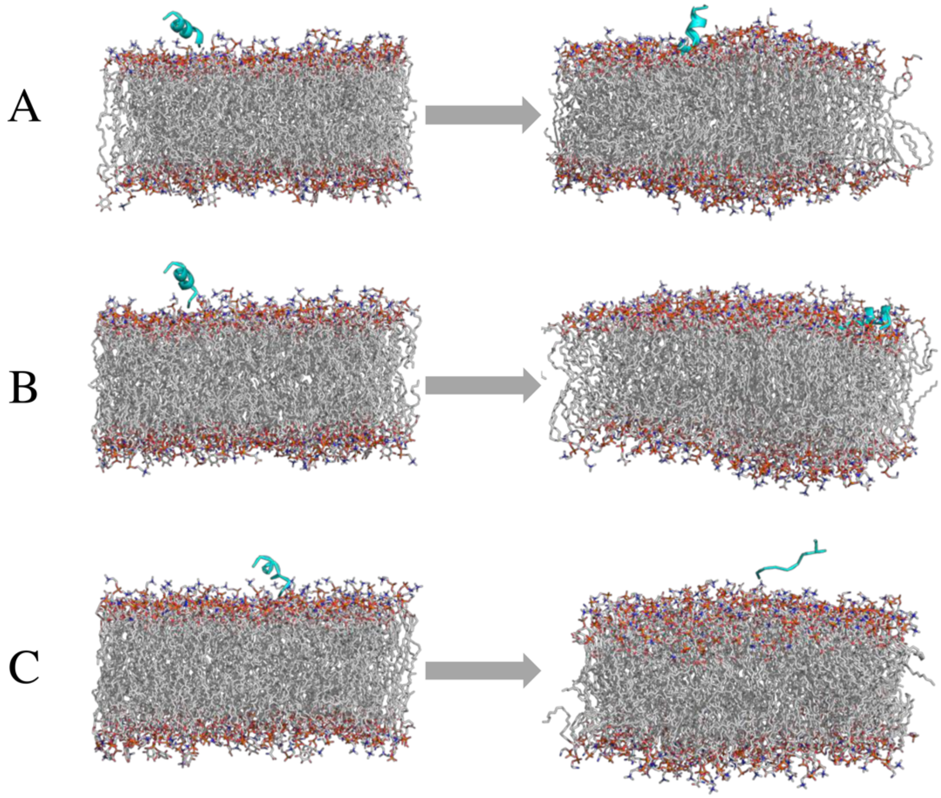

Supplementary Figure 20 | First and last molecular dynamics snapshots of peptides in the modelled membrane, illustrating penetration and potential disruption of the bilayer structure. A) peptide LLQWLLKRLKAK, B) peptide IILKKLLDFILK, and C) peptide TLLTAIVKLFLK. The lipid membrane is colored with carbon in white, oxygen in red, nitrogen in blue, and phosphate atoms in orange. The peptides are depicted in cyan.

`

Supplementary Table 8 | MD simulations checklist

Checklist Item	Yes/No	Where Addressed in Manuscript
**1. Convergence of Simulations and Analysis**		
**1a.** Is an evaluation presented in the text to show that the property being measured has equilibrated in the simulations (e.g., time-course analysis)?	Yes	In the Results and SI (Molecular Dynamics Experiments), we show time-course plots that demonstrate equilibration: CG RMSD/time (Suppl. Supplementary Fig 19E–F) and AA peptide–membrane contact evolution (Fig. 9D).
**1b.** Is it described in the text how simulations are split into equilibration and production runs and how much data were analyzed from production runs?	Yes	In SI (Molecular Dynamics Experiments): CG-MD used energy minimization NVT (250 ps) followed by NPT (500 ps) with position restraints, then 100 ns production; only production was analyzed. AA-MD ran 1 µs per system; the first 50 ns were discarded in plots and production data were analyzed.
**1c.** Are there at least 3 simulations per simulation condition with statistical analysis?	Yes	Both CG (100 ns each, 3 replicas) and AA (1 µs each, 3 replicas) per peptide. Statistics are reported across replicas (mean ± SD shading in Fig. 9D; replicate boxplots in Supplementary Fig 19-E).
**1d.** Is evidence provided in the text that the simulation results presented are independent of initial configuration?	Yes	Replicates were initialized independently (random placement relative to the bilayer in CG; independent runs in AA), and convergence/behavior is consistent across replicas (Supplementary Fig 19E–F; Fig. 9D).
**2. Connection to Experiments**		
**2a.** Are calculations provided that can connect to experiments (e.g., binding assays, J-couplings, etc.)?	Yes	We connect simulated membrane engagement to in vitro cytotoxicity: viability screen and IC_{50}s (Fig 7–8; Supplementary Fig 21). Mastoparan is included as a comparator; standard assay controls are used.
**3. Method Choice**		
**3a.** Do simulations contain membranes, membrane proteins, intrinsically disordered proteins, glycans, nucleic acids, polymers, or cryptic ligand binding?	Yes	Membrane bilayers with peptides were simulated. Compositions are specified (CG: POPC:POPS 1:1; AA: POPC:POPE:POPS:SM 3:2:1:1) in SI.
**3b.** Is it described in the text whether the accuracy of the chosen model(s) is sufficient for the question(s) under investigation (e.g., all-atom vs.	Yes	SI specifies MARTINI 2.2 for CG screening-scale behavior and AA simulations with CHARMM-GUI membranes and Desmond (standard OPLS/CHARMM settings) for mechanistic detail, consistent with the study goals.

`

coarse-grained, fixed charge vs. polarizable FF, etc.)?		
**3c.** Is the timescale of the event(s) under investigation beyond the brute-force MD timescale, requiring enhanced sampling?	No	Membrane insertion/engagement occurs within the simulated windows (CG equilibrates by ~50 ns; AA contacts grow over ~200–300 ns and are tracked to 1 µs), so enhanced sampling was not necessary (Supplementary Fig 19E–F; Fig. 9D).
**4. Code and Reproducibility**		
**4a.** Is a table provided describing the system setup (box dimensions, total atom count, water molecules, salt concentration, lipid composition)?	Yes	Detailed MD setup (box sizes, lipid composition, solvent/ions, thermostats/barostats) is reported in SI—Molecular Dynamics Experiments for CG-MD and AA-MD.
**4b.** Is it described what simulation and analysis software and versions are used?	Yes	GROMACS 2021 (CG), Schrödinger Desmond 2023 (AA), MDAnalysis 2.9.0 for analysis; visualization with VMD 1.9.4a53 /PyMOL 3.0.3; assay analysis with GraphPad Prism noted alongside experimental methods.
**4c.** Are other parameters for the system setup described (protonation states, structural restraints, nonbonded cutoff, thermostat/barostat, etc.)?	Yes	SI lists pH 7.0 PrepWizard protonation/minimization, position restraints during equilibration, V-rescale thermostat, Parrinello–Rahman barostat, and 1.2 nm real-space cutoffs for CG (with CG-appropriate settings).
**4d.** Are initial coordinate and simulation input files and a coordinate file of the final output provided as supplementary files or in a public repository?	Yes	We have deposited .gro, .top and .mae input files, along with final structures (for the AA); available in the public Zenodo repository: DOI: 10.5281/zenodo.17225962
**4e.** Is there custom code or custom force field parameters?	No	No custom force-field parameters were used; standard FFs were employed (MARTINI 2.2, CHARMM36, OPLS-4 in AA).

Supplementary Note 8: Invitro Validation

Selection of ACP candidates from PDB and GEN libraries

Experimental validation of ACPs is costly and resource-intensive, which explains why many generative modeling studies, despite promising computational results, lack experimental confirmation. Szymczak et al. evaluated 13 generative modeling tools for antimicrobial peptide (AMP) discovery and found that five did not include experimental validation[69]. Moreover, none of

`

the methods provided full access to code and data, posing reproducibility challenges. For ACPs, experimental validation is even rarer, highlighting a critical gap in generative modeling research.

Beyond the expense of peptide synthesis and testing, a major limitation is the absence of reliable intermediary methods to efficiently assess the therapeutic potential of newly generated sequences. In small-molecule drug discovery, molecular docking serves as a widely accepted proxy for estimating binding affinity and guiding experimental prioritization. However, ACPs, especially those with membranolytic mechanisms lack an equivalent in silico screening tool to predict experimental success. Given this challenge, virtual screening was prioritized to enhance confidence in candidate selection before committing to costly wet-lab validation. The PDB libraries leveraged sequences derived from known protein structures, where biological context and functional annotations provided additional confidence in their potential as ACPs (section "*In silico* validation of discovered anticancer peptide motifs", "Resonance between discovered motifs and known ACPs: Insights from PDB metadata" in Appendix F4). In contrast, the GEN library consisted of purely computationally generated sequences with no prior biological grounding. Without robust in silico proxies for assessing activity, experimentally validating a large fraction of GEN-derived peptides would have significantly increased the risk of failure, making the study financially and logistically impractical. Notably, Yue et al.[70] screened 3.8 million sequences from UniProt but only 100,000 from a generative model, reflecting similar challenges in prioritizing AI-generated peptides for experimental validation. Similarly, in this study, top 95 sequences from the PDB libraries and top 10 sequences from the GEN library were selected for in vitro validation.

This selection strategy does not imply that the generative model failed to produce active sequences but rather reflects a pragmatic approach to experimental resource allocation. The generative model was trained to produce peptides with physicochemical properties resembling helices, yet its ability to generate effective ACPs remains an open question. By focusing experimental validation on PDB-derived sequences while including a small but representative subset from the GEN pot, this study maximized the likelihood of identifying functional ACPs while still exploring the potential of generative modeling approaches.

Wet-lab characterization of 105 peptides SPOT-synthesized on cellulose membranes

`

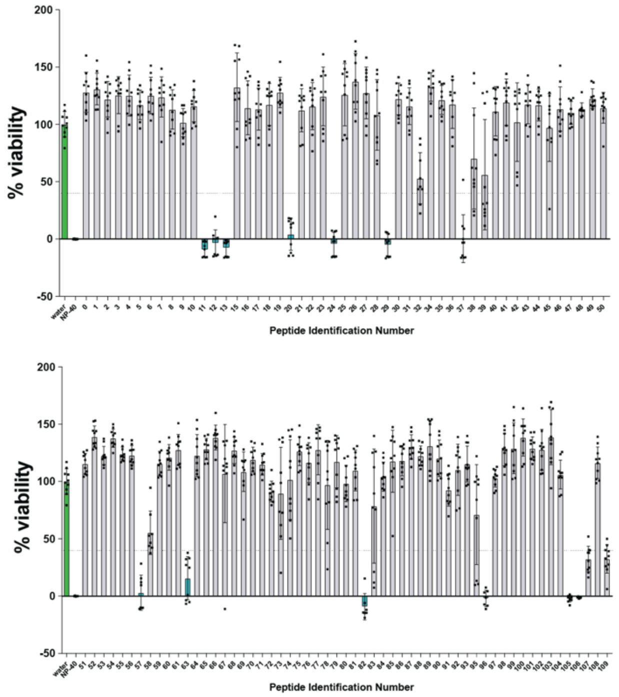

Peptide #	Sequence
11	LLQWLLKRLLKAK
12	KYLKALFKLALK
13	LLKALFKALKRL
20	LLKTLKWLMAS
24	LYIILKKLLDFI
29	IILKKLLDFILK
37	LLYLMRMWKAL
57	TLILTAVKLFLK
63	LIKCLLKSYIL
82	LLFLKSFQFLIK
96	LWLARFLKLFIAT
105	FLWKLKLKVIH
106	VLWKLVKKLLAA
107	SLWKLILLIAKH
109	LLFWIKLLAKS

Supplementary Figure 21 | Viability screen on MDA-MB-231 cells. 105 peptides were tested at 25 µM in triple negative MDA-MB-231 cells using the PrestoBlue assay for viability. Top peptides (in blue) with more than 60% inhibition of viability were selected for further analysis. dH_2O was used as the negative control (in green) and 1% NP-40 as the positive control. The data is from three biological replicates, each with four technical replicates.

`

Cytotoxicity of anti-cancer peptides on breast cancer and normal cells

The cytotoxicity of ACPs was assessed using both breast cancer and normal cells to evaluate their specificity and potential off-target effects. Experimental validation showed that 15 of the 105 synthesized peptides exhibited significant anti-cancer activity during the initial screen of SPOT-synthesized peptide samples. Subsequently, the top three peptides (11, 29, and 57) were further examined in viability assays that included normal breast cell line MCF10A and breast cancer cell lines MDA-MB-231, MCF7 and TamR3.

Certain phospholipids, such as phosphatidylserine (PS), are distinctly present on the outer leaflet of cancer cell membranes, unlike in normal cells where PS is confined to the inner leaflet[71]. This externalization of PS in cancer cells is a key factor that distinguishes them from normal cells and facilitates the selective binding and cytotoxicity of cationic peptides. Despite this difference, both cancer and normal breast cell types share a comparable lipid composition, primarily involving phosphatidylcholines (PCs), phosphatidylethanolamines (PEs), Phosphatidylinositols (PIs), and sphingomyelins (SMs)[72]. Mass spectrometry profiling between normal breast cell line (MCF10A) and breast cancer cell lines revealed approximate lipid ratios of 1:1.5 for PC(32:1), 1.5:1 for SM(34:0), and 2:1 for PI(38:4) (MCF10A:breast cancer cells)[72]. These ratios indicate that while there are differences, the overall lipid composition remains comparable, potentially contributing to the off-target cytotoxicity observed in normal breast cells.

In contrast, the membrane lipid compositions of PBMCs and RBCs differ significantly from those of breast cells. RBC membranes consist predominantly of PSs and PIs localized on the inner monolayer, while PCs and SMs are present on the outer monolayer, contributing to the membrane's structural asymmetry and fluidity[73]. PBMC membranes exhibit unique lipid compositions involving polyunsaturated fatty acids (PUFAs) and cholesterol, influencing membrane fluidity and permeability which are distinct from breast cells[74]. These differential lipid profiles may partially account for the relative resistance of PBMCs and RBCs to the cytotoxic effects of the peptides.

To refine these peptides for enhanced specificity towards cancerous breast cells, further modifications could be made to increase their affinity for lipid components uniquely upregulated in breast cancer cells. For instance, targeting specific lipid markers such as certain ceramides or glycosphingolipids, which are often elevated in malignant cancers, could improve selective cytotoxicity[75]. Incorporating peptide engineering strategies, such as enhancing amphipathic properties or incorporating specific binding domains for breast cancer cell-specific receptors, could also contribute to higher selectivity and reduced off-target effects.

Selection, Optimization, and Validation of Anticancer Peptides

The primary objective of this study was the discovery of novel ACPs. However, the selection process inherently favored membranotropic, cationic helical peptides, as this structural and physicochemical profile is well-documented in the literature as a hallmark of many known ACPs. This includes Mastoparan, on which the model training was based. While the identified ACPs represent promising candidates, further optimization through rational design and chemical modifications is necessary. The key contribution of this study lies in the development of an end-

`

to-end ACP discovery pipeline, which not only generates experimentally validated hits but also provides a high-quality dataset for data-driven ACP research. DL has yet to be fully leveraged in the field due to the scarcity of reliable data, and this study addresses that gap by presenting a structured computational-experimental framework.

To assess the cancer cell specificity of the selected ACPs while minimizing toxicity to healthy cells, experimental validation was performed using three distinct cell lines: MDA-MB-231, RBCs, and PBMCs. This selection was guided by prior work from Hilchie et al.[76], which demonstrated Mastoparan's selective cytotoxicity toward MDA-MB-231 relative to PBMCs. The findings of this study align with those observations, supporting the hypothesis that membranotropic ACPs differentiate cancerous from non-cancerous cells based on membrane composition differences, including increased anionic lipid content and altered membrane fluidity in cancer cells. Comparable studies in machine learning-driven ACP design, such as the work by Grisoni et al[21], have similarly validated selectivity using MCF-7 breast cancer cells and healthy human erythrocytes. While these cell lines provide a reasonable initial model system, incorporating additional cancer cell lines in future studies will allow for broader generalization of the findings.

The study's search strategy employed a systematic screening process that reduced an initial peptide space of 1.6 billion candidates through multiple filtering steps. Peptides were prioritized based on predicted anticancer potential, physicochemical properties, and structural compatibility with known ACP motifs. Pre-filtering based on physicochemical and structural properties is a common strategy in bioactive peptide screening. For instance, a recent study by Huang et al.[77] screened hexapeptides for antimicrobial activity by reducing an initial search space of 64 million sequences to a candidate pool of 3.93 million sequences using structural constraints associated with antimicrobial peptides. Similarly, in this study, helical peptides—recognized as a hallmark of many ACPs—were prioritized, systematically narrowing the search space to 36 million helical sequences from the PDB. The final three experimentally validated ACPs emerged from this stringent selection process. While the absolute number of validated hits may seem small relative to the initial search space, high attrition rates are expected in drug discovery pipelines, particularly for membrane-disruptive ACPs, which require a precise balance between activity and selectivity to avoid off-target cytotoxicity. Similar DL-driven peptide discovery studies have reported single-digit final peptide candidates, including Huang et al.[77] (three antimicrobial peptides), HydrAMP[69] (nine peptides), Das et al.[19] (two peptides), and Zhang et al.[78] (three peptides). In the context of ACP discovery, DL-driven studies with experimental validation remain scarce, highlighting the significance of this work.

The experimental hit rate of 14% (15 out of 105 candidates) further underscores the robustness of the computational pipeline. In peptide-based drug discovery, hit rates from large screening pools are typically low, with many studies reporting rates below 0.01%. Only a fraction of identified hits exhibit potency within a therapeutically relevant range. The high-throughput screening (HTS) validation in this study applied a stringent success criterion, classifying peptides as ACP candidates only if they exhibited >60% inhibition of viability in spot-array validation. In contrast, prior studies, such as those by Yue et al.[70] and Ma et al.[79], employed a much lower threshold (>20% inhibition), which, if applied to this study, would have increased the reported hit rate to 22 out of 105 candidates. Furthermore, Ma et al. evaluated peptides across 12 different cancer cell lines and classified a peptide as "active" if it showed activity against at least one cell line. This broader

`

classification unsurprisingly resulted in a higher reported hit rate. However, when specifically considering MDA-MB-231—a highly aggressive and treatment-resistant triple-negative breast cancer (TNBC) cell line—only 3 out of 40 peptides (7.5%) in their study exhibited activity. These peptides also displayed cytotoxicity toward control human cell lines, indicating a lack of selective anticancer activity. Given the challenges of targeting MDA-MB-231 cells, the decision to use this cell line for primary screening further strengthens this study's conclusions.

The potency of the identified ACPs, measured through IC50 values against MDA-MB-231 cells, ranged from 7.8 to 23.9 µM, with two outliers at 41.3 µM and 93.1 µM. While lower IC50 values are generally preferable, these results align with recent ACP literature. For example, Hilchie et al. reported an IC50 of 20–24 µM for a Mastoparan-derived ACP, while Law et al.[80] observed a similar range in their work. Additionally, Zakharova et al.[81] identified four selective ACPs with IC50 values between 5.0 and 8.2 µM after 72 hours of incubation. Results from more recent studies are listed in Supplementary Table 9. Many peptide-based drugs achieve strong in vivo efficacy despite IC50 values in the low-to-mid micromolar range, owing to factors such as targeted delivery, protease resistance, and improved bioavailability. Hence, this study's IC50 values fall within an expected range for ACPs, further supporting the therapeutic potential of the identified candidates.

Supplementary Table 9 | IC50 range of most promising candidates reported in recent studies.

**Cancer Cell Line(s)**	**IC50 range (µM)**	**#peptides**	**Reference**	**Comments**	**Year**
MDA-MB231	20–24	1	Hilchie et. al[76]	After 24 h incubation	2017
B16F10, BxPC-3, K562, PANC-1, HeLa, HCT-116, MIA-PaCa-2, 4T1, HepG2, U251, PC12, A549	3.91->50	10	Yue et. al[70]	After 36 h incubation	2025
MDA-MB231	7.39	1	Law et. al[80]	After 48h incubation. After 24h IC50 was 64.04µM	2023
MCF-7	35.34	1	Velayutham et al.[82]	IC50 value of 35.34 µM, at 24 h	2023
HepG2	18.75	1	Velayutham et al.[83]	After 24 hr, IC50 is 21µM and after 48 hr, 18.75µM	2022
HeLa, MCF7, MDA-MB-231	7.8-19	4	Zakharova et al.[81]	After 72 h incubation, & MCF-10A 11.7-19.5 uM	2022

`

MDA-MB-231, BT-20, BT-474, SKBR3	3.5->100	6	Oliveira et al.[84]	After 24 h incubation	2021
MDA-MB-231, MCF-7, TamR3	7.8- 25	15	Ours	After 24h incubation. 7.8μM to 23.9μM with 2 notable outliers with 41.3μM and 93.1μM.	2025

Selectivity Considerations in Anticancer Peptide Discovery

Anticancer selectivity in peptides is highly context-dependent, influenced by factors such as membrane composition, intracellular targets, proteolytic stability, and cellular uptake mechanisms. The heterogeneity of cancer cells in lipid profiles, receptor expression, and metabolic activity complicates the broad-spectrum classification of ACPs. For example, MDA-MB-231 cells exhibit a unique surface charge distribution due to increased phosphatidylserine exposure, making them more susceptible to cationic ACPs. In contrast, other cancer types, such as glioblastomas and leukemias, may exhibit distinct membrane fluidity, lipid organization, or resistance mechanisms that modulate ACP potency and selectivity. While hemolytic (erythrocyte lysis) and leucolytic (PBMC lysis) assays are commonly used to assess peptide selectivity, they do not comprehensively predict ACP activity across all cancer types, as normal blood cells possess membrane properties distinct from those of malignant cells.

Recognizing the limitations of selectivity testing in a single adherent cancer cell line, additional validation experiments were conducted to strengthen the conclusions. These experiments included MCF7 (ER+, low metastatic potential breast cancer), TamR3 (tamoxifen-resistant breast cancer), and MCF10A (non-tumorigenic mammary epithelial cells) alongside MDA-MB-231. This expanded evaluation provided a broader context for ACP activity, allowing for a more comprehensive assessment of therapeutic relevance. The findings suggest that ACP activity across MDA-MB-231, hemolytic, and leucolytic assays provides a useful framework for initial candidate selection; however, further refinements in peptide design and selectivity optimization remain necessary. Future work will address these refinements to improve therapeutic efficacy while minimizing off-target effects.

The classification of ACPs versus non-ACPs presents additional challenges due to dataset variability. The CancerPPD dataset, widely used for ACP classification, exhibits considerable heterogeneity in terms of cell line selection, assay conditions, and peptide concentrations. These inconsistencies can introduce systematic biases in model training, affecting the generalizability of predictions. While many studies have relied on broad ACP/non-ACP classification models derived from CancerPPD, the present study aimed to develop a more targeted approach by focusing on Mastoparan analogues with well-characterized experimental measurements for percentage inhibition in MDA-MB-231 cells. The CancerPPD classification model was not used to assess hemolytic activity against PBMCs, as its primary utility lay in the identification of generalized ACP features towards cancer cells.

`

$D_{Mastoparan}$ facilitated the development of a regression model optimized for ACP potency prediction specifically in MDA-MB-231 cells, offering a more reliable, cell-line-specific framework than broad classification models alone. While the study prioritized Mastoparan-based regression modeling, CancerPPD was not entirely excluded. ACPs across different cancer types share conserved physicochemical and structural features, including amphipathicity and net charge, which influence membrane disruption, secondary structure preferences such as α-helicity, which contribute to potency, and hydrophobicity, which affects peptide penetration into lipid bilayers. To integrate these shared properties, an ACP classification model trained on CancerPPD was combined with the MDA-MB-231 regression model in an ensemble approach. This strategy allowed the model to capture both broad-spectrum ACP characteristics and cell-line-specific potency predictions, enhancing overall predictive accuracy by leveraging both generalized and targeted molecular features.

Supplementary Note 9: TARSA

Ablation and Case Studies

Ablation

TARSA's reward function and hotspot selection criteria drove effective exploration of peptide libraries. Each module's importance was assessed through ablation tests. The reward components $\{r_{dist}, r_{oracle}, r_{penalty}\}$ were individually removed to evaluate their impact. Removing r_{oracle} limited exploration, causing the agent to primarily exploit nearby hotspots, aligning with studies that indicate intrinsic rewards promote state-space exploration (Supplementary Fig 22A). Removing r_{dist} eliminated the agent's convergence targets, resulting in failed trajectories and fewer discovered peptides, since the agent sometimes violated state-space bounds (Supplementary Fig 22B). Without $r_{penalty}$, episodes lasted until time budget exhaustion, increasing peptide discovery but not meaningfully reducing the state-space (Supplementary Fig 22C). Each reward component was crucial, as shown by a high hotspot-to-peptide ratio, indicating sampling from diverse activity cliffs in no ablation case (Supplementary Fig 22D). Hotspot selection remained constant across ablation experiments to isolate reward component effects. By default, a TARSA variant, κ-TARSA-P.S. was used, which showed most effective exploration and stability across random initializations (Supplementary Table 7).

Case studies

Extrinsic batch sizes were tested to understand their effect on the learning policy and screening effectiveness. Extrinsic batch sizes refer to the subsets of the peptide library used for training TARSA (B_{train}) or for parallelized offline inference (B_{samp}). The desired criteria were high cumulative and mean potency for the discovered peptides (Supplementary Fig 22E-G). B_{samp}: training with 2 million random samples and varying B_{samp} sizes, larger batches improved screening efficiency without compromising sample quality.

B_{train}: various sizes were tested with fixed B_{samp}= 2 million demonstrating that 250,000 peptides (smaller than the library) were effective, with B_{train}= B_{samp}=2 million also performing well.

B_{train}=B_{samp}: finally, we observed comparable performance between batch sizes of 100,000 and 2 million, reasonably satisfying our goals within 500,000 timesteps. To optimize resources, B_{train}=B_{samp}=2 million were chosen for rest of the study, reducing computational requirements.

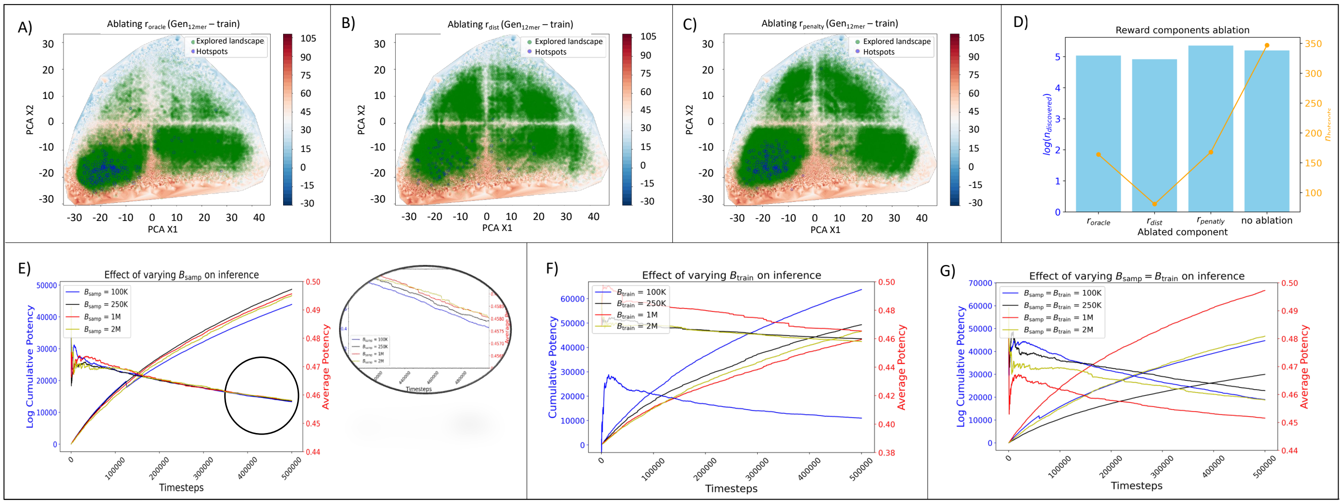

Supplementary Figure 22 | TARSA ablation and case studies. (A-D) Ablation: Effect of each reward component on TARSA's exploratory capabilities. (E-G) TARSA inference with different extrinsic batch sizes.

12-mer ACP discovery

In this study, to experimentally evaluate the cytotoxic potency of the top ACP candidates identified using the computational method described, the SPOT synthesis method was used to make peptides arrays on cellulose supports[85]. To ensure consistency between peptide samples on the array, a peptide sequence length of 12 residues was defined and all of the synthesized peptides were of uniform length (12-mer) across the array.

Screening PDB_{large}: TARSA screening of PDB_{large} with trained policy $\pi_{\theta 1}$ yielded 3.2M motifs with predicted cell-viability inhibition >40% (Supplementary Fig 23). Next, longer motifs (>12 residues) were truncated into 12-mers using a sliding window approach with a step size of one residue to preserve overlapping subsequences and maintain structural relevance. This ensured that any biologically significant sub-motifs remained intact. Shorter motifs (<12 residues) were extended by appending neighboring amino acids from their corresponding protein sequences in PDB_{large} to create 12-mer peptides. The resulting dataset, PDB_{12mer}, was then used in ACP-candidate search phase of the PepSce workflow to identify candidates for synthesis and validation.

Screening PDB_{12mer}: PDB_{12mer} followed a nearly identical distribution as the training dataset for policy trained on PDB_{large}-$\pi_{\theta 1}$ (Supplementary Fig 8). Consequently, $\pi_{\theta 1}$ was transferred to PDB_{12mer} for screening. Evaluating our protocol by running multiple instances of $\pi_{\theta 1}$ on PDB_{12mer},

`

we found a statistically significant difference in potency between discovered peptides and random peptides from PDB_{12mer} ($p < 0.001$) (Supplementary Fig 15). In comparison, similar to TARSA training for PDB_{large}, a fresh online policy $\pi_{\theta 2}$ trained directly on $PDB_{12\text{-}mer}$ discovers more diverse hotspots and hence diverse ACP candidates in comparison to offline inference using $\pi_{\theta 1}$ (Supplementary Fig 24). However, training a new policy is computationally expensive and we found that using offline policy $\pi_{\theta 1}$ yielded sufficient diversity and it was cost-effective at scale.

Screening GEN_{12mer}: Herein, it was demonstrated that even a naïve probabilistic generative model, combined with fast screening method TARSA, can discover potent candidates while significantly reducing search overhead. 30 million novel 12-mer peptides (GEN_{12mer}) were generated by sampling amino acids according to probabilities for helical peptides[1]. New policy $\pi_{\theta 2}$ was trained on 2 million random samples of GEN_{12mer}, and then used for inference on remaining GEN_{12mer}. On a randomly chosen subset of 2 million generated peptides, the first-k discovered peptides had similar mean potency as the top-k discovered peptides (Fig.5A-C). This indicated that TARSA prioritizes exploitation in early stages and discovers most potent candidates first. Through TARSA screening of GEN_{12mer}, 3.5 million peptides with an average predicted potency of 44.8% cell inhibition were discovered, reducing the search space by 92.5%.

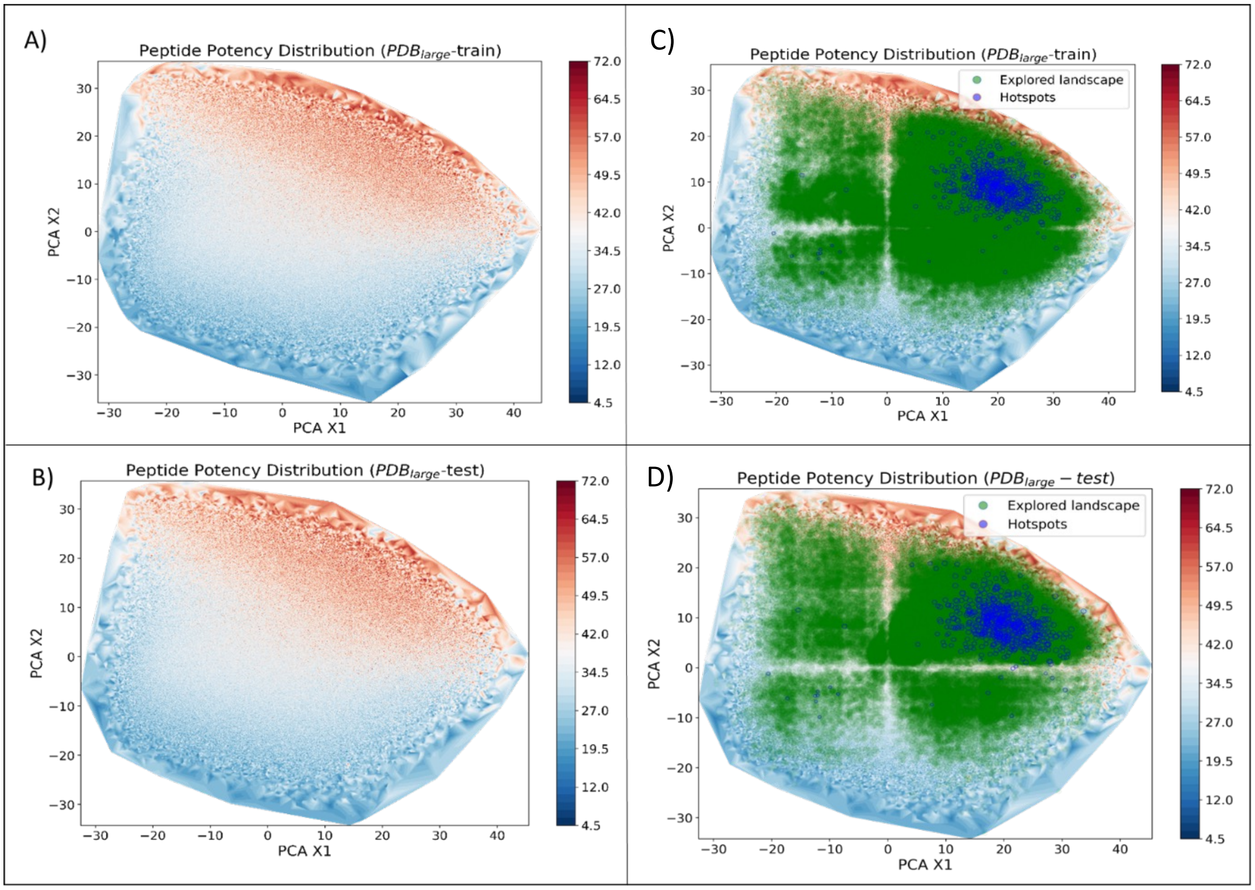

Supplementary Figure 23 | Screening results for PDB$_{large}$ library across the first two Eigen vectors, showing TARSA's exploration of high-potency regions and avoidance of low-potency regions. (A, C) 2 million random peptides sampled from PDB$_{large}$, used for training and corresponding explored peptides (green) by application of TARSA. (C, D) A random subset of PDB$_{large}$ used for batched rollout of trained policy.

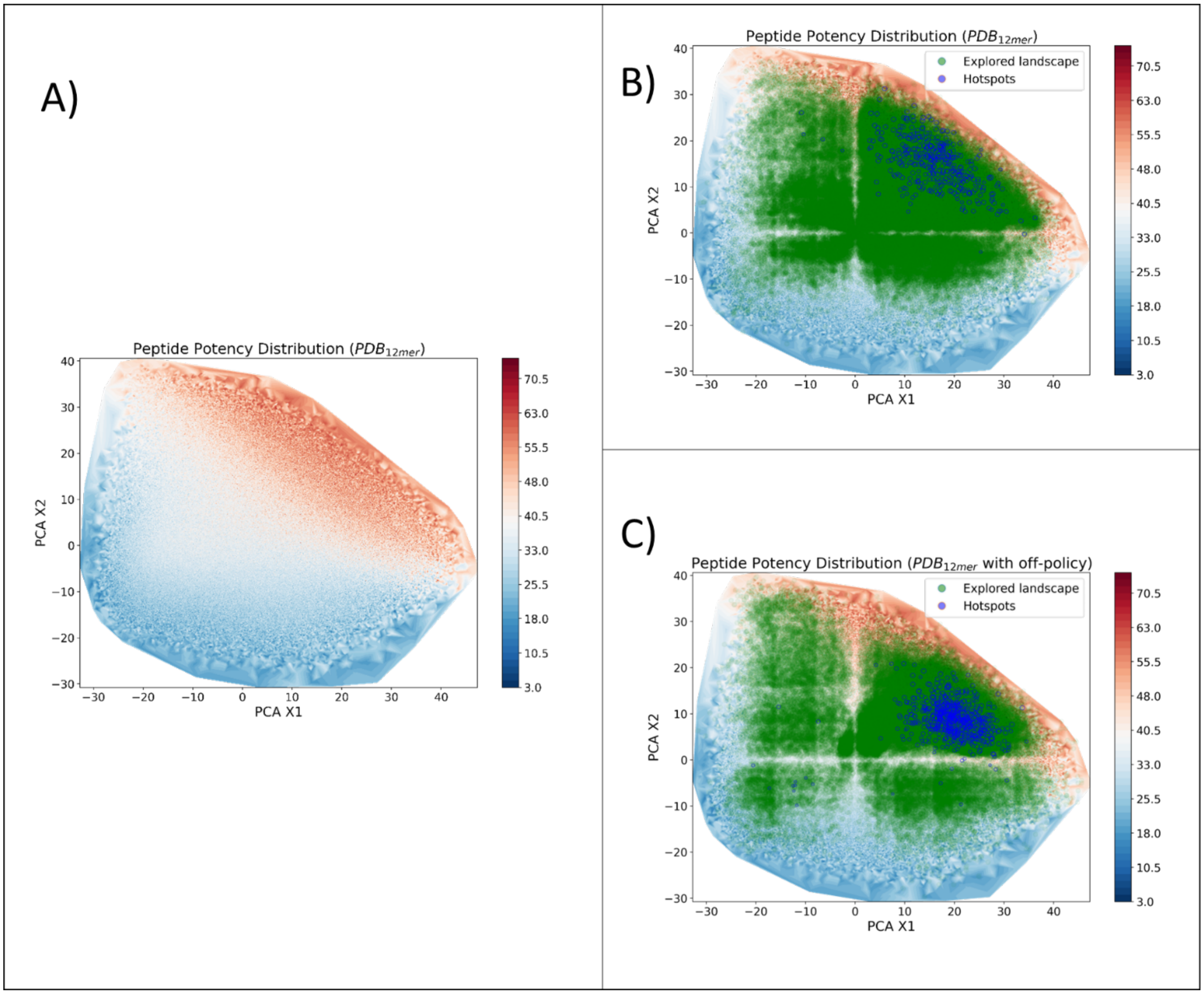

Supplementary Figure 24 | Comparison of PDB$_{12mer}$ screening with an online and offline trained TARSA policy. (A) PDB$_{12mer}$ with predicted percentage cell inhibition towards MDA-MB231 cells. (B) Roll out of trained policy on PDB$_{12mer}$ on same dataset (C) Roll out of policy trained on PDB$_{large}$ dataset.

Supplementary Method 1:

In this section, the problem of HTVS is formulated as a constrained optimization problem, curated datasets to perform peptide screening are described, and an in-depth explanation of the proposed RL method is provided. All computational experiments were run on a computing cluster with four Tesla V100-SXM2 GPUs, with 32GB RAM each.

`

Problem Formulation

While searching through large libraries for effective therapeutic candidates, the focus is on finding the smallest subset with most diverse and potent samples, thereby reducing the search space and the need for exorbitant compute. This objective is specified as the following constrained optimization problem:

$$\text{Objective} : \max_{s \subseteq S} \left[\frac{Div(s) \times (\sum_s f(s))}{|s|} \right] \quad (5)$$

Subject to constraint : $Q(\boldsymbol{s}) \leq C$

where, $f(.)$ is an expensive black-box estimator of potency.

Q($\mathbf{s}$) is the total number of queries made to $f(.)$ in search for a potent and diverse candidate set and C is the available budget to query $f(.)$

This specification is computationally intractable. Moreover $f(.)$ is assumed as an ML approximator of the potency function. We propose to utilize RL to approximate the discrete optimization objective with continuous control.

Dataset

This work relies on two broad categories of data – (i) labeled datasets recording peptide activity toward cancer cells, (ii) unlabeled peptide libraries for ACP screening. To this end, two labeled inhouse datasets were sourced – Mastoparan MDA-MB231 and Mastoparan PBMC. Mastoparan MDA-MB231 and Mastoparan PBMC consist of Mastoparan derivatives with single amino acid substitution and report percentage cell inhibition on TNBC cells (MDA-MB-231) and cytotoxicity towards healthy human PBMC respectively at 12.5 μM concentration. While the former dataset was used to train the regression model (Oracle proxy- f_θ^o) to predict peptide potency, the later dataset was used in the filtering phase of PepSce workflow. CancerPPD is the largest publicly available dataset of experimentally validated ACPs containing 3317 entries. Upon eliminating cyclic peptides, D-form, and mixed peptides and dropping peptides with missing experimental values, 590 ACPs were left. One limitation of the CancerPPD dataset is that it lacks experimentally verified non-ACPs, hindering the development of a reliable ACP prediction model. To this end, other researchers have used surrogate negative samples from antimicrobial peptides that have no anti-cancer activity[86,87]. Here, 590 negative samples were randomly sampled from these studies to create a balanced binary ACP dataset. A classifier trained on this dataset was utilized to predict the activity of hits discovered in PepSce.

Next, two unlabelled datasets were curated from PDB to screen for potent ACPs. To accomplish this, we extracted all helices from the crystallographic structure of every protein in the downloadable PDB (accessed Aug 2022). At this stage, only amino acid sequences were considered, without incorporating terminal charges. An extensive peptide library (PDBlarge) was created by employing a sliding window approach to divide helices into peptide sequences of length l ($5 < l \leq 15$). Likewise, we constructed a selective peptide library of fixed sequence length 12-

`

mers (PDB_{12mer}) by considering most potent helical ACP motifs from PDB_{large}. Longer helical sequences were divided into multiple 12-lengthed peptides using a sliding window approach. For shorter motifs, the PDB was searched and neighboring amino acids were included, until the resulting sequence was a 12-mer peptide.

Finally, in order to demonstrate the utility of PepSce on artificially generated peptides, the Gen_{12mer} dataset was constructed by sampling from precomputed probabilities of helical ACPs. Table 1 summarizes the different datasets used in this work.

Learning the Oracle proxy

In ML, an oracle is a hypothetical perfect model that can provide the correct label for any instance with zero error. In most cases, there is a high cost associated with querying the oracle and hence getting the oracle's output for all instances in a very large dataset would be extremely expensive. In this work, an oracle proxy was defined, that takes the form of a regression model to predict peptide potency (percentage cell inhibition) on breast cancer cells (MDA-MB231) at 12.5 μM concentration. For training the oracle proxy, the Mastoparan dataset was utilized.

Feature Engineering: The Mastoparan dataset consisted of fixed-length (14) amino acid sequences. Several conventional and DL representation methods[88] were explored to vectorize these sequences. Conventional methods include the translation of peptide sequences to a fixed length numerical representation using descriptors (features)[32,89–91]. Each of these encodings has been shown to capture biological relationships and high-level conformational information from amino acid sequences, hence the choice of the right encoding is non-trivial. Here, two of these descriptors (modlamp and ifeatures) that have been specifically reported to be successful for bioactive peptide prediction tasks[19,20] were aggregated.

DL method: Under the same configuration of a 3-layered MLP and a random forest model, usefulness of the 3 descriptors (modlamp, ifeat, and ESM) was accessed by training a regression model for each setting. The results were 5-fold cross validated to evaluate model performance. The 2 best performing descriptors - modlamp and ifeature were chosen to further train the Oracle proxy. To this end, a high-level fusion model was trained on the aforementioned peptide descriptors. The modlamp and ifeat descriptors were processed with a 3-layered MLP and a Convolutional Neural Network respectively. The shared representations learned from these 2 submodules were then fused together to predict the peptide bioactivity regression output. As is typical with experimental data, the peptide density on either end of the cytotoxicity spectrum is low.

Since the correct prediction of such peptides is more rewarding than the correct prediction of moderately toxic ones, a density-based weighted mean squared error loss, equation 1, was utilized based on the ideas from Steininger et al[92]. A larger gradient from the rare peptide samples ensures better model estimates for such peptides in addition to the common ones.

$$\mathcal{L}(\hat{y}, y) = \frac{1}{N}\sum_{i=1}^{N} f_w(\alpha, y_i)\|y_i - \hat{y}\|_2^2 \tag{6}$$

`

where f_w is a weighting function that measures the rarity of the sample based on its kernel density estimate, and α is a hyperparameter that controls the model's ability to focus on rare (highly toxic) or common (moderately toxic to non-toxic) peptides.

Reinforcement Learning for goal-directed peptide discovery

This section elaborates the TARSA algorithm and the setup under which it screens peptides (Fig.1). TARSA operates on a navigational board ($\mathcal{B}$) containing 2D PCA projections of peptides. It trains the policy π that an RL agent follows to explore $\mathcal{B}_{train}$. The trained policy is then rolled out on several other navigational boards $\mathcal{B}_{eval}$ corresponding to subsets of a large peptide library.

Low dimensional representation of peptides

To effectively represent peptides on a navigational board, and to learn a deep RL navigational policy, it is imperative to employ computationally efficient and biochemically meaningful representations of peptides. In this study, representations derived from ESM models were created (Appendix C2) to fulfill this purpose.

Deep RL relies on state representations that are low-dimensional, evolve through time, are influenced by the agent's actions, and sufficiently characterize the system to predict its future[93]. While recent advances in deep RL have shown that policies may be learned directly from raw observations[94,95], it is beneficial for RL algorithms to take advantage of low dimensional representations for faster and better convergence of training [96,97]. To this end, ESM provides dense and compact representations for peptide sequences. These embeddings, however, are relatively high-dimensional (768). The use of high dimensional ESM representations in their native form may slow down policy training. Since speed is paramount in screening tasks and RL policies converge faster in low-dimensional state space, a 2-dimensional PCA projection of ESM representation was adopted to define our state space. Moreover, such a reduced representation enables us to overcome the problem of sparsity associated with searching for peptides in a high-dimensional space.

Target Adaptive Reinforcement Learning for Sampling Activity landscape

In RL, Markov Decision Processes (MDPs) serve as a foundational framework for modeling the sequential decision-making problems. A MDP is defined by a tuple (S, A, P, R, γ), where 'S' represents the set of states, 'A' denotes the set of actions, 'P' defines the transition probabilities between states upon taking actions, 'R' signifies the reward function, and 'γ' is the discount factor for future rewards. In this formalism, agents interact with an environment by selecting actions based on their current state, transitioning to new states according to the dynamics of the system, receiving rewards based on their actions and states, and aiming to maximize the cumulative reward over time. The state-action space ($\mathcal{S} \times \mathcal{A}$) encapsulates all possible combinations of states and actions that the agent can encounter during its decision-making process, providing a structured representation of the environment's dynamics and guiding the agent's exploration and exploitation strategies towards achieving optimal outcomes.

In this work, the RL agent learns to navigate the state space defined by 2-dimensional PCA projections of peptides and learns an optimal policy ($\pi_{\psi,\phi}$) to navigate to hotspots where the

`

average peptide potency levels are high. This enables efficient *in silico* screening of large peptide-libraries while balancing the exploration-exploitation tradeoff.

The states, actions, rewards, and the environment in which TARSA operates are now described.

Environment: Environment is an abstraction of the world in which the agent lives and performs actions. The environment in this work is modelled as a 2-dimensional *"navigation board* $\mathcal{B}$*"*. The peptides to be screened reside on $\mathcal{B}$ defined by their corresponding first two principal components. The agent samples actions from a learned policy that guides its traversal of $\mathcal{B}$. As the policy rolls out, the agent attempts to reach a dynamically updated goal called *target hotspot* (h_{target}) on $\mathcal{B}$. At the beginning of each episode, the agent is initialized at a random location within the environment. As the agent moves through the environment, at each location within $\mathcal{B}$, it encounters one of 4 possibilities – {*blank, peptide, goal, revisit*}. Based on the nature of the location, a reward is awarded to the agent. "*Blank*" represents the absence of peptides at the agent's location ($\boldsymbol{X}_t^a$), $r_{oracle} = 0$; "*peptide*" corresponds to cases where a peptide is present at $\boldsymbol{X}_t^a$, $r_t = \beta_i * r_i$; "*goal*" is when $X_t^a = h_{target,t}$, a bonus reward is awarded for successful episode completion; finally, in "*revisit*" cases X_t^a has already been explored within current episode, $r_{oracle} = 0$, $r_{dist} = 0$. Locations on $\mathcal{B}$ corresponding to peptides of sufficiently high potency are considered hotspots. Through many episodes of training, the agent finds several such hotspots with progressively increasing potency. An episode terminates when one of the following 3 conditions is met – (i) the agent steps out of bounds of $\mathcal{B}$, (ii) a more potent peptide is discovered (h_{target} is updated) or (iii) after 1,000 steps.

State: $\boldsymbol{s}_t = (\boldsymbol{X}_t^a, d_t^{ag}, \boldsymbol{X}_t^g)$ consists of the agent's location $\boldsymbol{X}_t^a = (x_{1,t}^a, x_{2,t}^a)$ where $x_{1,t}^a$ and $x_{1,t}^a$ are the Cartesian coordinates along the first and second principal components respectively at timestep *t*. Similarly, $\boldsymbol{X}_t^{(g)}$ denotes dynamic goal ($h_{target,t}$) location at time *t*, and $d_t = ||X_t^a - X_t^g||_2$ is the Euclidean distance between the agent's current position and current goal ($h_{target,t}$).

Action: $\boldsymbol{a}_t = \{\lambda_t, \theta_t\}$, where $\lambda_t \in [0.95, 1.05]$ and $\theta_t \in [-\frac{\pi}{3}, \frac{\pi}{3}]$ are step size and direction defining the agent's movement in the environment at timestep *t*.

State transitions are deterministic in this environment i.e. $P_{sa} = 1 \; \forall \boldsymbol{s} \in \mathcal{S}, \boldsymbol{a} \in \mathcal{A}$. An action $\boldsymbol{a}_t = \{\lambda_t, \theta_t\}$ moves the agent $\boldsymbol{X}_t^a \xrightarrow{a_t} \boldsymbol{X}_{t+1}^a$ as $\boldsymbol{X}_{t+1}^a = \lambda_t \times [\boldsymbol{R}_{\theta_t}^T \boldsymbol{X}_t^a]$. R_{θ_t} is the rotation matrix defined as $\boldsymbol{R}_{\theta_t} = \begin{bmatrix} \cos\theta_t & -\sin\theta_t \\ \sin\theta_t & \cos\theta_t \end{bmatrix}$.

Reward Shaping to Drive Exploration: The agent performs guided traversal in Cartesian coordinates on $\mathcal{B}$ with a carefully designed reward function r_t.

$$r(\boldsymbol{s}_t, \boldsymbol{a}_t) = \sum_{i=1}^{3} \beta_i r_i^{(t)} \tag{7}$$

where, $r_i \in \{r_{dist}, r_{oracle}, r_{penalty}\}$ and β_i is a hyperparameter controlling the strength of each reward component. Each reward component is now described in detail.

Reward Function

The agent performs guided traversal in Cartesian coordinates on $\mathcal{B}$ guided by the following reward function r_t.

`

$$r(\boldsymbol{s}_t, \boldsymbol{a}_t) = \beta_1 r_{dist} + B_2 r_{oracle} + \beta_3 r_{penatly} \quad (8)$$

where the β_i are hyperparameters controlling the importance of each reward component. Each reward component is now described in detail.

r_{dist} is the distance component of the reward function, which promotes convergence of the agent to $h_{target,t}$ on $\mathcal{B}$ and is defined as $1 - \left(\frac{d(s_t, h_{target})}{d(s_0, h_{target}) + \epsilon}\right)^{\frac{1}{2}}$, where $d(.)$ is the Euclidean distance, and $\epsilon \rightarrow 0$ maintains numerical stability during the computation. $r_{oracle} = f_{\Phi}(s_t)$ is peptide potency predicted by the Oracle proxy. Finally, $r_{penalty}$ is a small penalty for the number of steps that ensures that the agent takes the shortest path to reach h_{target}.

Psychologists have identified intrinsic motivation or curiosity as a key driver for individuals to engage in activities for inherent satisfaction rather than external rewards, enhancing learning by motivating exploration and knowledge acquisition. Similarly, in RL, intrinsic rewards become useful when extrinsic rewards are unavailable or sparse[98]. Intrinsic rewards are primarily used to encourage the agent to explore "novel" states[99–101] i.e., the integration of intrinsic rewards plays a pivotal role in promoting exploration within vast state spaces. By designing intrinsic rewards that incentivize agents to navigate through uncharted territories effectively, exploration in complex environments is enhanced. These rewards are crafted to encourage the discovery of novel states and paths that lead to valuable outcomes, leading to a deeper understanding of the environment and maximizing the chances of uncovering optimal solutions. In this work, the r_{oracle} reward serves a similar purpose. It encourages the agent to follow a path that maximizes the likelihood of encountering highly active peptides on its way to h_{target}, thereby promoting the exploration of the chemical space and increasing the chances of finding potent hits. This also ensures that the agent is not stuck in a local optimum with a large number of moderately potent peptides but rather chooses few but highly active peptides.

Together, these three reward components enable the agent to quickly converge to the regions of high activity in the peptide latent space while collecting several highly active peptides on the way. Since there can be multiple trajectories through which h_{target} can be reached, the agent is not awarded a positive reward for re-exploring the same state within an episode. This also prevents the agent from getting trapped in local optima and encourages it to explore different trajectories. Furthermore, a large negative penalty is imposed on the agent for taking actions that lead to the violation of legal constraints of $\mathcal{B}$. The legal constraints are set as min(PCA(X)), max(PCA(X)), where X is the library to be screened.

Stochastic hotspot sampling driven exploration

In RL, adapting to dynamically changing goals offers opportunities for flexible and adaptive decision-making. By incorporating mechanisms to handle shifting objectives, RL agents can effectively navigate environments where goals evolve over time. Techniques such as goal-conditioned RL (GCRL) [102], meta-learning[103], or hierarchical RL[104,105] enable agents to adjust their strategies in response to changing objectives, allowing for robust performance in dynamic settings. In this work, GCRL setting is used. Since in GCRL, the goals to be achieved by the agent are explicitly given, it does not have to discover these goals. Moreover, since we use the goal

`

representation directly in the GCRL formulation, it is more sample efficient in learning a policy that maximizes coverage of the chemical space[106].

Posterior Sampling for Target Hotspot Estimation

As a way of choosing and dynamically updating h_{target} from a set of candidate targets (H_c), posterior sampling is utilized. For any hotspot $h_i \in \boldsymbol{H}_c$, it is assumed that the corresponding associated rewards (agent's rewards when h_i is selected as h_{target}) are drawn from a Gaussian distribution parameterized as $\mathcal{N}(\mu_i, \sigma_i)$. The posteriors of these parameters can inform the RL-agent about the regions with high average peptide potency.

The likelihood function $P(\boldsymbol{R_i} \mid \mu_i, \sigma_i)$ represents the probability of observing rewards $\boldsymbol{R_i}$ given the parameters μ_i and σ_i.

$$P(\boldsymbol{R_i} \mid \mu_i, \sigma_i) = \prod_{j=1}^{N} \frac{1}{\sqrt{2\pi}\sigma_i} exp\left(-\frac{(\boldsymbol{R}_{ij}-\mu_i)^2}{2\sigma_i^i}\right) \quad (9)$$

Here, R_{ij} represents the j-th reward observed for hotspot h_i, and N is the total number of rewards collected for hotspot h_i.

To this end, the posterior of μ_i (P($\mu_i \mid R_i$)) and σ_i (P($\sigma_i \mid R_i$)) is estimated by collecting rewards R_i from $\pi(\phi,\psi; h_i)$. π is a policy characterized by actor (ϕ) and critic (ψ) networks respectively when its h_{target} is established at h_i and collects rewards (R_i) for N_{samp} timesteps.

Applying Bayes rule, the posterior distributions for μ_i and σ_i can be calculated as:

$$\begin{aligned} P(\mu_i \mid \boldsymbol{R_i}) &\propto P(\boldsymbol{R_i} \mid \mu_i, \sigma_i) \times P(\mu_i) \\ P(\sigma_i \mid \boldsymbol{R_i}) &\propto P(\boldsymbol{R_i} \mid \mu_i, \sigma_i) \times P(\sigma_i) \end{aligned} \quad (10)$$

where $P(\mu_i)$ and $P(\sigma_i)$ are the prior distributions for μ_i and σ_i respectively.

A MCMC method is used to sample from the posterior distributions $P(\mu_i|R_i)$ and $P(\sigma_i|\mathbf{R_i})$. This involves constructing Markov chains whose stationary distributions are the desired posterior distributions as described next.

The h_{target} sampling proceeds via first initializing a candidate h_{target}, $h_0 \in \mathcal{X}, \mu_0, \sigma_0 \sim P(\mu_i), P(\sigma_i)$. At sampling timesteps $t \ mod \ N_{samp} = 0$, a new sample μ_i and σ_i are sampled from the proposal distribution as $\mu_i \sim Q(\mu_i \mid \mu_{i-1})$, $\sigma_i \sim Q(\sigma_i \mid \sigma_{i-1})$, such that μ_{i-1} and σ_{i-1} are the current parameter estimates for likelihood $P(\mathbf{R} \mid \mu, \sigma)$. N is a hyperparameter controlling the frequency of h_{target} updates. Since MCMC updates maybe slow, N_{samp} is chosen to be sufficiently large such that it allows for stable policy training without significantly burdening policy training. $N_{samp} = 50$ worked reasonably well, empirically. The proposed estimates μ_i and σ_i are accepted according to probability α defined as

$$\alpha = min\left(1, \frac{P(\boldsymbol{R_i}|\mu_i, \sigma_i).P(\mu_i).P(\sigma_i).Q(\mu_{i-1}|\mu_i).Q(\sigma_{i-1}|\sigma_i)}{P(\boldsymbol{R_i}|\mu_i, \sigma_i).P(\mu_i).P(\sigma_i).Q(\mu_{i-1}|\mu_i).Q(\sigma_{i-1}|\sigma_i)}\right) \quad (11)$$

`

This ensures that the acceptance probability is bounded by $\alpha \in [0,1]$. The proposed estimates are then accepted or rejected as

$$\mu_i = \begin{cases} \mu_i \; with\; probability\; \alpha \\ \mu_{i-1} \; with\; probability\; 1-\alpha \end{cases} \quad (12)$$

$$\sigma_i = \begin{cases} \sigma_i \; with\; probability\; \alpha \\ \sigma_{i-1} \; with\; probability\; 1-\alpha \end{cases} \quad (13)$$

When these Markov chains are trained sufficiently long, the chains can be assumed to have reached stationary distribution and the resulting parameter estimates will converge to the target distribution $P(\boldsymbol{R_i} \mid \mu_i, \sigma_i)$.

Following the estimation of the posterior distribution, posterior samples $\mu_i \sim P(\mu_i \mid \mathbf{R_i})$ and $\sigma_i \sim P(\sigma_i \mid R_i)$ are drawn. The new h_{target} is then determined by sampling a Gaussian distribution parametrized as $\mathcal{N}(\mu_i, \sigma_i)$ corresponding to each $h_i \in H_c$.th

$$h_{target} = \underset{i}{argmax}\,(X_1, X_2, ..., X_n) \quad (14)$$

$$\text{where, } X_i \sim \mathcal{N}(\mu_i, \sigma_i)$$

$$\text{and } \mu_i = \mathbb{E}[\mu_i \mid \mathbf{R_i}]$$

$$\sigma_i = \mathbb{E}[\sigma_i \mid \mathbf{R_i}]$$

Refer to algorithm 3 (appendix E) for details of our hotspot updating procedure.

Policy updates with dynamic goals:

Following Schulman et al.[107], PPO is adopted to learn the agent's policy. The dynamic goals and the proposed reward structure modify the policy update rule. The new Advantage function now incorporates the MCMC sampled h_{target}. Specifically,

$$A_{t,\pi_\phi}^{h_{target}} = Q_{\pi_\phi}^{h_{target}}(\boldsymbol{s}, \boldsymbol{a}\,; \boldsymbol{\psi}) - V_{\pi_\phi}^{h_{target}}(\boldsymbol{s}\,; \boldsymbol{\psi}) \quad (15)$$

where, $Q_{\boldsymbol{\psi}}(\boldsymbol{s}_t, \boldsymbol{a}_t)$is the action-value function (critic), which estimates the expected cumulative reward obtained by taking action a_t in state s_t, parameterized by an MLP $\boldsymbol{\psi}$. Similarly, $V_{\boldsymbol{\psi}}(\boldsymbol{s}_t)$ is the value function (critic), which estimates the expected cumulative reward obtained from state s_t, parameterized by the same MLP $\boldsymbol{\psi}$. Under the composite reward structure, $Q_{\boldsymbol{\psi}}(.)$ and $V_{\boldsymbol{\psi}}(.)$ become

$$Q_{\pi_\phi}^{h_{target}}(\boldsymbol{s}_t, \boldsymbol{a}_t\,; \boldsymbol{\psi}) = \mathbb{E}_{\pi_\phi}\left[\sum_{k=0}^{\infty} \gamma^k \left(\sum_{i=1}^{3} \beta_i r_i^{(t+k)}(\boldsymbol{s}_t, \boldsymbol{h_{target}})\right) \mid \boldsymbol{s}_t, \boldsymbol{a}_t; \boldsymbol{\psi}\right] \quad (16)$$

`

$$V_{\pi_{\phi}}^{h_{target}}(\boldsymbol{s}_t\,;\boldsymbol{\psi}) = \mathbb{E}_{\pi_{\phi}}\left[\sum_{k=0}^{\infty}\gamma^k\left(\sum_{i=1}^{3}\beta_i r_i^{(t+k)}(\boldsymbol{s}_t,\boldsymbol{h_{target}})\right) \mid \boldsymbol{s}_t;\boldsymbol{\psi}\right] \tag{17}$$

Now, the new Advantage can be written as

$$A_{t,\pi_{\phi}}^{h_{target}} = \sum_{i=1}^{3}\beta_i r_i^{(t)}(\boldsymbol{s}_t,\boldsymbol{h_{target}}) + \gamma V_{\pi_{\phi}}^{h_{target}}(\boldsymbol{s}_{t+1}\,;\boldsymbol{\psi}) - V_{\pi_{\phi}}^{h_{target}}(\boldsymbol{s}_t\,;\boldsymbol{\psi}) \tag{18}$$

This definition of $A_{t,\pi_{\phi}}^{h_{target}}$ and surrogate objective $\mathcal{L}^{CLIP}$ of PPO leads to our policy training objective

$$J(\boldsymbol{\phi}) = \mathbb{E}_{\pi_{\phi}}\left[\mathcal{L}^{CLIP}(\boldsymbol{\phi}) - c.\left[H(\pi_{\boldsymbol{\phi}}(.\mid \boldsymbol{s}))\right]\right] \tag{19}$$

such that

$$\mathcal{L}^{CLIP}(\boldsymbol{\phi}) = \mathbb{E}_{\pi_{\phi}}\left[min\left(\frac{\pi_{\phi}(a_t|s_t)}{\pi_{\phi_{old}}(a_t|s_t)}.A_{t,\pi_{\phi}}^{\boldsymbol{h_{target}}},\ clip\left(\frac{\pi_{\phi}(a_t|s_t)}{\pi_{\phi_{old}}(a_t|s_t)},1-\epsilon,1+\epsilon\right).A_{t,\pi_{\phi}}^{\boldsymbol{h_{target}}}\right)\right] \tag{20}$$

and entropy $H(\pi_{\boldsymbol{\phi}}(.\mid \boldsymbol{s}_t)) = -\mathbb{E}_{\pi_{\phi}}\left[\sum_{a\in\mathcal{A}}\pi_{\phi}(\boldsymbol{a}\mid \boldsymbol{s}_t)\log\pi_{\boldsymbol{\phi}}(\boldsymbol{a}\mid \boldsymbol{s}_t)\right]$

TARSA & Variants: Efficient *In Silico* Screening of Peptides

An informative and generalizable representation, such as ESM, ensures that peptides with similar structural and biochemical properties are close to each other in the latent space. It is reasonable to assume that such similar peptides may have similar toxicity levels toward the cancer cells. There may be several regions of the latent space formed by similar and putatively bioactive peptides where the average peptide potency levels would be high. Such regions are called *"bioactive hotspots"*(h_{target}). These *hotspots* are not known to the agent apriori. The objective for the agent is 3-fold - (i) discover such hotspots in the low-dimensional projection of the peptide latent space- $\mathcal{B}$, (ii) learn an optimal policy ($\pi_{\psi,\phi}$) to navigate to hotspots, and (iii) identify high potency peptides in the neighborhood of these hotspots. To meet these objectives, a deep RL based algorithm – TARSA is designed, that learns to navigate on a 2D PCA projection of the peptide representation and enables efficient *in silico* screening of large peptide-libraries. Three variants of the TARSA algorithm are described here - (i) *greedy*-TARSA (ii) TARSA P.S. and (iii) κ-TARSA P.S. which differ primarily in their approach towards hotspot selection.

An explorative strategy enables the agent to learn multiple solution trajectories for the same optimization problem. Conceptually, random initialization and bioactive hotspots-guided explorations by TARSA are analogous to training students to solve the same problem from multiple viewpoints, thereby exposing them to different techniques to build their general problem-solving abilities. For *greedy*-TARSA, the hotspot - h_{target} is greedily updated to the location of the maximal potency peptide encountered by the agent thus far, while the posterior sampling variants of TARSA (TARSA P.S., κ-TARSA P.S.) employ posterior sampling to determine h_{target} from a candidate hotspot set, H_c.

TARSA P.S. builds upon *greedy*–TARSA by progressively adding the location of the maximum potency peptide encountered so far into H_c. κ–TARSA P.S., on the other hand, extends H_c by including the location of all encountered peptides with a potency within a factor of κ : $\kappa \leq 1$ from the maximum potency peptide explored thus far by the agent. For an episode, TARSA P.S. and κ-

`

TARSA P.S. treat the problem of sampling h_{target} from the candidate set H_c as a multi-armed bandit problem where each candidate h_{target} is considered a bandit. The selection of h_{target} from within H_c set is performed using posterior sampling and is defined in next section. Formally, TARSA P.S. is a special case of κ−TARSA P.S. for $\kappa = 1$ i.e. κ−TARSA P.S. generalizes TARSA P.S. by incorporating additional information about the location of highly potent peptides, but when κ is set to 1, the additional information is not used, and κ−TARSA P.S. reduces to TARSA P.S. This dynamic target update strategy ensures maximal coverage of the chemical space by the agent. The support provided by a larger number of hotspot candidates facilitates the exploration of diverse regions of peptide chemical space by exploiting spatial correlations among peptides with similar potencies and hence improving the efficiency of the search process. Refer to Supplementary Table 6,7 for a summary of the TARSA variants.

The agent is tasked with discovering several hotspots that may be present in the 2-dimensional peptide latent space which is modeled as a navigation board $\mathcal{B}$. This is achieved by first initializing a random position on $\mathcal{B}$ as a dummy hotspot. Hotspots serve as suggested goals for the agent to converge. At the beginning of each episode, the agent is initialized at random starting positions. At any time t, continuous actions of a certain step size (λ) and direction (θ) are sampled from the current policy $\pi_{\psi,\phi}(\lambda_t,\theta_t \mid s_t)$. Using the sampled actions, the agent's position and therefore its internal state are updated thereby transporting the agent to a new location (as defined in the state transitions above). Through the span of an episode, the agent explores several positions on $\mathcal{B}$ and investigates peptides at these locations by querying the Oracle proxy. The policy parameters are updated to drive the agent closer to the selected hotspot $h_{target,t}$ by maximizing the expected total reward over time. The details of training for TARSA are presented in algorithm 1, appendix E.

Inference phase using policy transfer

Once the agent has learned a policy π on a subset of the peptide library, S, rollouts from π are obtained to screen the remainder of the peptide library $D-\{S\}$ (Fig.1B). To do so, the agent is initialized at random locations on the PCA-projection spanned by $D - \{S\}$. The h_{target} for the rollout of each trajectory is determined as $h_{target} = \text{argmax}_i(X_1, X_2, ..., X_n)$ where $X_i \sim N(\mu_i, \sigma_i^2)\ \forall i \in H_c$. During the inference phase, The parameters μ_i and σ_i are not estimated through posterior sampling (algorithm 3, appendix E). Instead, the estimates of these parameters that were learned during the training phase are used. Employing such a hotspot selection scheme allows a greater level of exploitation in the neighborhood of hotspots exhibiting a higher mean potency. Finally, several rollouts are carried out on $D - \{S\}$, and the peptides encountered by the agent are collected as ACP candidates for subsequent screening (algorithm 3). On each batch, the screening is continued until the average potency of discovered peptides drops below 40% cell inhibition or until the termination criteria are met (Appendix F2). For screening GEN_{12mer}, the trained policy $\pi_{\theta 3}$ was rolled out on 14 disjoint instances of 2M samples each. Full inference on GEN_{12mer} was run until top-7.5% peptides were discovered from each evaluation batch. This resulted in roughly 150,000 peptides with a mean potency of 45.2% cell inhibition. A parallelized batched rollout inference was performed on 14 Tesla V100 GPUs with five independent policy rollouts on each batch for speed. The entire screening took five days.

Metrics

The mean potency, novelty, and diversity together define the usefulness of any sampler as a screening agent. Since the training data for oracle proxy consists of single amino-acid substitutions of parent Mastoparan, we define novelty of the sampled set as the average distances of the

`

candidates from the Mastoparan sequence. Finally, diversity is the average Levenshtein distance between candidates in the sampled set. First-k and Top-k are defined as the first "k" samples from a sampler trained to convergence, while top-k are "k" samples with maximal scores after sampling for a fixed time budget. In experiments, each model was sampled for 1 hour to calculate top-k performance. These metrics were used to compare our method against baseline methods (Tab.1)

$$Mean(S) = \frac{\sum_{i=1}^{|S|} f^{\theta}(s_i)}{|S|} \quad (21)$$

$$Novelty = \frac{\sum_{i=1}^{|S|} Lev(s_{mastoparan}, \ s_i)}{|S|} \quad (22)$$

$$Diversity = \frac{\sum_{i=1}^{|S|} \sum_{j=1, j \neq i}^{|S|} Lev(s_i, s_j)}{|S|(|S|-1)} \quad (23)$$

$$Hit\ Rate = \frac{hits_A}{|N|} \times 100 \quad (24)$$

where, $s_i, s_j \in S$ and S is the sampled set of candidate peptides, $f^{\theta}(.)$ is the oracle proxy. $Lev(.)$ is the Levenshtein distance. $hits_A$ are the number of discovered/generated peptides with predicted % cell inhibition > 50% and |N| = 50,000.

Consensus Filtering and Cytotoxicity Ranking

As a means of orthogonal evaluation of the peptides discovered by PepSce workflow, the CancerPPD dataset[35] is utilized. To this end, a binary classifier is designed to distinguish ACPs from non-ACPs. Architecturally, the regression head of the surrogate oracle proxy is modified with a binary classification head and the binary cross-entropy loss is used for training (Fig 2C). For cytotoxicity prediction, we utilize the same classifier architecture and toxinpred data provided by[108]. Once the cytotoxic peptides selected by TARSA were filtered out using the classification model, the remaining peptides were ranked in order of their predicted %-cell inhibition of PBMC cells. To this purpose, the architecture employed was same as as surrogate oracle proxy with Mastoparan-PBMC dataset for training. Both classification and regression models were compared against random forest and XGBoost baseline models. The details of architectural design and hyperparameters are provided in appendix.D2-4. ACP candidates with predicted %-cell inhibition of PBMC cells at 12.5uM < 40% were considered as non-toxic and hence suitable for subsequent *in vitro* studies.

SUPPLEMENTARY REFERENCES

1. Gabernet, G., Müller, A. T., Hiss, J. A. & Schneider, G. Membranolytic anticancer peptides. *Medchemcomm* **7**, 2232–2245 (2016).
2. Chiangjong, W., Chutipongtanate, S. & Hongeng, S. Anticancer peptide: Physicochemical property, functional aspect and trend in clinical application (Review). *Int J Oncol* **57**, 678–696 (2020).
3. Huang, Y. *et al.* Role of helicity of α-helical antimicrobial peptides to improve specificity. *Protein Cell* **5**, 631–642 (2014).

`

4. Cock, P. J. A. *et al.* Biopython: freely available Python tools for computational molecular biology and bioinformatics. *Bioinformatics* **25**, 1422–1423 (2009).
5. De Souza, B. M., Dos Santos Cabrera, M. P., Neto, J. R. & Palma, M. S. Investigating the effect of different positioning of lysine residues along the peptide chain of mastoparans for their secondary structures and biological activities. *Amino Acids* **40**, 77–90 (2011).
6. Douglas, S., Hoskin, D. W. & Hilchie, A. L. Assessment of Antimicrobial (Host Defense) Peptides as Anti-Cancer Agents. *Methods in Molecular Biology* **1088**, 159–170 (2014).
7. Giangaspero, A., Sandri, L. & Tossi, A. Amphipathic α helical antimicrobial peptides. *European Journal of Biochemistry* **268**, 5589–5600 (2001).
8. Sani, M. A. & Separovic, F. How Membrane-Active Peptides Get into Lipid Membranes. *Accounts of Chemical Research* **49**, 1130–1138 (2016).
9. Jin, Y. *et al.* Antimicrobial activities and structures of two linear cationic peptide families with various amphipathic β-sheet and α-helical potentials. *Antimicrobial Agents and Chemotherapy* **49**, 4957–4964 (2005).
10. Jones, D. T. Protein secondary structure prediction based on position-specific scoring matrices. *Journal of molecular biology* **292**, 195–202 (1999).
11. Cuff, J. A. & Barton, G. J. Application of multiple sequence alignment profiles to improve protein secondary structure prediction. *Proteins* **40**, 502–511 (2000).
12. Montgomerie, S. *et al.* PROTEUS2: a web server for comprehensive protein structure prediction and structure-based annotation. *Nucleic acids research* **36**, (2008).
13. Zavrtanik, U., Lah, J. & Hadži, S. Estimation of Peptide Helicity from Circular Dichroism Using the Ensemble Model. *Journal of Physical Chemistry B* **128**, 2652–2663 (2024).
14. Forood, B., Feliciano, E. J. & Nambiar, K. P. Stabilization of alpha-helical structures in short peptides via end capping. *Proceedings of the National Academy of Sciences* **90**, 838–842 (1993).
15. Wang, D., Chen, K., Kulp, J. L. & Arora, P. S. Evaluation of biologically relevant short α-helices stabilized by a main-chain hydrogen-bond surrogate. *Journal of the American Chemical Society* **128**, 9248–9256 (2006).
16. Abramson, J. *et al.* Accurate structure prediction of biomolecular interactions with AlphaFold 3. *Nature 2024 630:8016* **630**, 493–500 (2024).
17. Drozdetskiy, A., Cole, C., Procter, J. & Barton, G. J. JPred4: a protein secondary structure prediction server. *Nucleic Acids Research* **43**, W389–W394 (2015).
18. Yan, J. *et al.* Deep-AmPEP30: Improve Short Antimicrobial Peptides Prediction with Deep Learning. *Molecular Therapy. Nucleic Acids* **20**, 882 (2020).
19. Das, P. *et al.* Accelerated antimicrobial discovery via deep generative models and molecular dynamics simulations. *Nat Biomed Eng* **5**, 613–623 (2021).
20. Chen, J., Cheong, H. H. & Siu, S. W. I. xDeep-AcPEP: deep learning method for anticancer peptide activity prediction based on convolutional neural network and multitask learning. *J Chem Inf Model* **61**, 3789–3803 (2021).
21. Grisoni, F. *et al.* Designing anticancer peptides by constructive machine learning. *ChemMedChem* **13**, 1300–1302 (2018).

`

22. Yang, L. *et al.* Accelerating the discovery of anticancer peptides targeting lung and breast cancers with the Wasserstein autoencoder model and PSO algorithm. *Brief Bioinform* **23**, 1–11 (2022).
23. Cho, K. *et al.* Learning Phrase Representations using RNN Encoder-Decoder for Statistical Machine Translation. *EMNLP 2014 - 2014 Conference on Empirical Methods in Natural Language Processing, Proceedings of the Conference* 1724–1734 (2014) doi:10.3115/v1/d14-1179.
24. Bateman, A. *et al.* UniProt: the universal protein knowledgebase. *Nucleic Acids Res* **45**, D158–D169 (2017).
25. Rao, R., Meier, J., Sercu, T., Ovchinnikov, S. & Rives, A. Transformer protein language models are unsupervised structure learners. *bioRxiv* 2020.12.15.422761 (2020) doi:10.1101/2020.12.15.422761.
26. Rives, A. *et al.* Biological structure and function emerge from scaling unsupervised learning to 250 million protein sequences. *Proc Natl Acad Sci U S A* **118**, e2016239118 (2021).
27. Vaswani, A. *et al.* Attention Is All You Need. *Advances in Neural Information Processing Systems* **2017-December**, 5999–6009 (2017).
28. Kawashima, S. *et al.* AAindex: amino acid index database, progress report 2008. *Nucleic Acids Research* **36**, D202–D205 (2008).
29. Lee, T. Y., Chen, S. A., Hung, H. Y. & Ou, Y. Y. Incorporating Distant Sequence Features and Radial Basis Function Networks to Identify Ubiquitin Conjugation Sites. *PLOS ONE* **6**, e17331 (2011).
30. Sandberg, M., Eriksson, L., Jonsson, J., Sjöström, M. & Wold, S. New chemical descriptors relevant for the design of biologically active peptides. A multivariate characterization of 87 amino acids. *Journal of Medicinal Chemistry* **41**, 2481–2491 (1998).
31. Ioffe, S. & Szegedy, C. Batch Normalization: Accelerating Deep Network Training by Reducing Internal Covariate Shift. *32nd International Conference on Machine Learning, ICML 2015* **1**, 448–456 (2015).
32. Cherkasov, A. & Jankovic, B. Application of 'inductive'QSAR descriptors for quantification of antibacterial activity of cationic polypeptides. *Molecules* **9**, 1034–1052 (2004).
33. Barley, M. H., Turner, N. J. & Goodacre, R. Improved Descriptors for the Quantitative Structure-Activity Relationship Modeling of Peptides and Proteins. *Journal of Chemical Information and Modeling* **58**, 234–243 (2018).
34. Zhou, P. *et al.* Systematic Comparison and Comprehensive Evaluation of 80 Amino Acid Descriptors in Peptide QSAR Modeling. *Journal of Chemical Information and Modeling* **61**, 1718–1731 (2021).
35. Tyagi, A. *et al.* CancerPPD: a database of anticancer peptides and proteins. *Nucleic Acids Res* **43**, D837–D843 (2015).
36. Saravanan, V. & Lakshmi, P. T. V. ACPP: A web server for prediction and design of anti-cancer peptides. *Int J Pept Res Ther* **21**, 99–106 (2015).
37. Sharma, N., Naorem, L. D., Jain, S. & Raghava, G. P. S. ToxinPred2: an improved method for predicting toxicity of proteins. *Briefings in bioinformatics* **23**, (2022).

`

38. Bateman, A. *et al.* UniProt: the universal protein knowledgebase. *Nucleic Acids Research* **45**, D158–D169 (2017).
39. Bairoch, A. & Apweiler, R. The SWISS-PROT protein sequence database and its supplement TrEMBL in 2000. *Nucleic acids research* **28**, 45–48 (2000).
40. Shoombuatong, W., Schaduangrat, N. & Nantasenamat, C. Unraveling the bioactivity of anticancer peptides as deduced from machine learning. *EXCLI J* **17**, 734–752 (2018).
41. Wu, J.-M. *et al.* Structure and function of a custom anticancer peptide, CB1a. *Peptides (N.Y.)* **30**, 839–848 (2009).
42. Ghaly, G. *et al.* Anti-Cancer Peptides: Status and Future Prospects. *Molecules 2023, Vol. 28, Page 1148* **28**, 1148 (2023).
43. Gifford, J. L., Hunter, H. N. & Vogel, H. J. Lactoferricin: A lactoferrin-derived peptide with antimicrobial, antiviral, antitumor and immunological properties. *Cellular and Molecular Life Sciences* **62**, 2588–2598 (2005).
44. Imai, K. & Mitaku, S. Mechanisms of secondary structure breakers in soluble proteins. *Biophysics* **1**, 55 (2005).
45. Pace, C. N. & Scholtz, J. M. A Helix Propensity Scale Based on Experimental Studies of Peptides and Proteins. *Biophysical Journal* **75**, 422–427 (1998).
46. Garvie, C. W. *et al.* Structure of PDE3A-SLFN12 complex reveals requirements for activation of SLFN12 RNase. *Nature Communications 2021 12:1* **12**, 1–16 (2021).
47. Han, K. *et al.* The NEDD8-activating enzyme inhibitor MLN4924 induces G2 arrest and apoptosis in T-cell acute lymphoblastic leukemia. *Oncotarget* **7**, (2016).
48. D\'\iaz-Gómez, J. L., Castorena-Torres, F., Preciado-Ortiz, R. E. & Garc\'\ia-Lara, S. Anti-cancer activity of maize bioactive peptides. *Frontiers in chemistry* **5**, 44 (2017).
49. Cutone, A. *et al.* Lactoferrin's Anti-Cancer Properties: Safety, Selectivity, and Wide Range of Action. *Biomolecules 2020, Vol. 10, Page 456* **10**, 456 (2020).
50. Zhang, Y., Lima, C. F. & Rodrigues, L. R. In vitro evaluation of bovine lactoferrin potential as an anticancer agent. *International Dairy Journal* **40**, 6–15 (2015).
51. Uggerhøj, L. E. *et al.* Rational Design of Alpha-Helical Antimicrobial Peptides: Do's and Don'ts. *ChemBioChem* **16**, 242–253 (2015).
52. Gaspar, D., Veiga, A. S. & Castanho, M. A. R. B. From antimicrobial to anticancer peptides. A review. *Front Microbiol* **4**, 294 (2013).
53. Singh, M. *et al.* Computational Design of Biologically Active Anticancer Peptides and Their Interactions with Heterogeneous POPC/POPS Lipid Membranes. *Journal of Chemical Information and Modeling* **60**, 332–341 (2020).
54. Marrink, S. J. & Tieleman, D. P. Perspective on the Martini model. *Chemical Society Reviews* **42**, 6801–6822 (2013).
55. Tozzini, V. Coarse-grained models for proteins. *Current Opinion in Structural Biology* **15**, 144–150 (2005).
56. De Jong, D. H. *et al.* Improved parameters for the martini coarse-grained protein force field. *Journal of Chemical Theory and Computation* **9**, 687–697 (2013).
57. Tien, M. Z., Sydykova, D. K., Meyer, A. G. & Wilke, C. O. Peptidebuilder: A simple python library to generate model peptides. *PeerJ* **2013**, e80 (2013).

`

58. Madhavi Sastry, G., Adzhigirey, M., Day, T., Annabhimoju, R. & Sherman, W. Protein and ligand preparation: parameters, protocols, and influence on virtual screening enrichments. *Journal of computer-aided molecular design* **27**, 221–234 (2013).
59. Marrink, S. J., Risselada, H. J., Yefimov, S., Tieleman, D. P. & De Vries, A. H. The MARTINI Force Field: Coarse Grained Model for Biomolecular Simulations. *Journal of Physical Chemistry B* **111**, 7812–7824 (2007).
60. Wassenaar, T. A., Ingólfsson, H. I., Böckmann, R. A., Tieleman, D. P. & Marrink, S. J. Computational lipidomics with insane: A versatile tool for generating custom membranes for molecular simulations. *Journal of Chemical Theory and Computation* **11**, 2144–2155 (2015).
61. Welcome to the GROMACS documentation! — GROMACS 2021 documentation. https://manual.gromacs.org/2021/index.html.
62. McGibbon, R. T. *et al.* MDTraj: A Modern Open Library for the Analysis of Molecular Dynamics Trajectories. *Biophysical journal* **109**, 1528–1532 (2015).
63. Humphrey, W., Dalke, A. & Schulten, K. VMD: Visual molecular dynamics. *Journal of Molecular Graphics* **14**, 33–38 (1996).
64. Kang, X. *et al.* DRAMP 2.0, an updated data repository of antimicrobial peptides. *Scientific Data 2019 6:1* **6**, 1–10 (2019).
65. Quemé-peña, M. *et al.* Membrane association modes of natural anticancer peptides: Mechanistic details on helicity, orientation and surface coverage. *International Journal of Molecular Sciences* **22**, 8613 (2021).
66. Jo, S., Kim, T., Iyer, V. G. & Im, W. CHARMM-GUI: A web-based graphical user interface for CHARMM. *Journal of Computational Chemistry* **29**, 1859–1865 (2008).
67. Preta, G. New Insights Into Targeting Membrane Lipids for Cancer Therapy. *Frontiers in Cell and Developmental Biology* **8**, 571237 (2020).
68. PyMOL | www.pymol.org. https://www.pymol.org/pymol.html?
69. Szymczak, P. *et al.* Discovering highly potent antimicrobial peptides with deep generative model HydrAMP. *Nature Communications 2023 14:1* **14**, 1–23 (2023).
70. Yue, J. *et al.* Discovery of anticancer peptides from natural and generated sequences using deep learning. *Int J Biol Macromol* **290**, 138880 (2025).
71. Kaynak, A. *et al.* Phosphatidylserine: The Unique Dual-Role Biomarker for Cancer Imaging and Therapy. *Cancers 2022, Vol. 14, Page 2536* **14**, 2536 (2022).
72. He, M., Guo, S. & Li, Z. In situ characterizing membrane lipid phenotype of breast cancer cells using mass spectrometry profiling. *Scientific Reports 2015 5:1* **5**, 1–13 (2015).
73. Mohandas, N. & Gallagher, P. G. Red cell membrane: past, present, and future. *Blood* **112**, 3939–3948 (2008).
74. Hon, G. M. *et al.* Peripheral blood mononuclear cell membrane fluidity and disease outcome in patients with multiple sclerosis. *Indian journal of hematology & blood transfusion : an official journal of Indian Society of Hematology and Blood Transfusion* **28**, 1–6 (2012).
75. Preta, G. New Insights Into Targeting Membrane Lipids for Cancer Therapy. *Frontiers in Cell and Developmental Biology* **8**, 571237 (2020).

`

76. Hilchie, A. L. *et al.* Mastoparan is a membranolytic anti-cancer peptide that works synergistically with gemcitabine in a mouse model of mammary carcinoma. *Biochimica Et Biophysica Acta (BBA)-Biomembranes* **1858**, 3195–3204 (2016).
77. Huang, J. *et al.* Identification of potent antimicrobial peptides via a machine-learning pipeline that mines the entire space of peptide sequences. *Nature Biomedical Engineering 2023 7:6* **7**, 797–810 (2023).
78. Zhang, J. *et al.* Large-scale screening of antifungal peptides based on quantitative structure–activity Relationship. *ACS Med Chem Lett* **13**, 99–104 (2021).
79. Ma, Y. *et al.* Efficient Mining of Anticancer Peptides from Gut Metagenome. *Advanced Science* **10**, 2300107 (2023).
80. Law, D. *et al.* In silico identification and in vitro assessment of a potential anti-breast cancer activity of antimicrobial peptide retrieved from the ATMP1 Anabas testudineus fish peptide. *PeerJ* **11**, e15651 (2023).
81. Zakharova, E., Orsi, M., Capecchi, A. & Reymond, J. L. Machine Learning Guided Discovery of Non-Hemolytic Membrane Disruptive Anticancer Peptides. *ChemMedChem* **17**, e202200291 (2022).
82. Velayutham, M. *et al.* Aquatic Peptide: The Potential Anti-Cancer and Anti-Microbial Activity of GE18 Derived from Pathogenic Fungus Aphanomyces invadans. *Molecules* **28**, 6746 (2023).
83. Velayutham, M. *et al.* Anti-Cancer and Anti-Inflammatory Activities of a Short Molecule, PS14 Derived from the Virulent Cellulose Binding Domain of Aphanomyces invadans, on Human Laryngeal Epithelial Cells and an In Vivo Zebrafish Embryo Model. *Molecules* **27**, 7333 (2022).
84. Oliveira, F. D. *et al.* The antimetastatic breast cancer activity of the viral protein-derived peptide vCPP2319 as revealed by cellular biomechanics. *The FEBS Journal* **289**, 1603–1624 (2022).
85. Hilpert, K., Winkler, D. F. H. & Hancock, R. E. W. Peptide arrays on cellulose support: SPOT synthesis, a time and cost efficient method for synthesis of large numbers of peptides in a parallel and addressable fashion. *Nat Protoc* **2**, 1333–1349 (2007).
86. Wu, X., Zeng, W., Lin, F., Xu, P. & Li, X. Anticancer Peptide prediction via multi-kernel cnn and attention model. *Frontiers in Genetics* **13**, (2022).
87. Ahmed, S. *et al.* ACP-MHCNN: an accurate multi-headed deep-convolutional neural network to predict anticancer peptides. *Sci Rep* **11**, 23676 (2021).
88. ElAbd, H. *et al.* Amino acid encoding for deep learning applications. *BMC bioinformatics* **21**, 1–14 (2020).
89. Müller, A. T., Gabernet, G., Hiss, J. A. & Schneider, G. modlAMP: Python for antimicrobial peptides. *Bioinformatics* **33**, 2753–2755 (2017).
90. Chen, Z. *et al.* iFeature: a python package and web server for features extraction and selection from protein and peptide sequences. *Bioinformatics* **34**, 2499–2502 (2018).
91. Chou, K.-C. Prediction of protein cellular attributes using pseudo-amino acid composition. *Proteins: Structure, Function, and Bioinformatics* **43**, 246–255 (2001).
92. Steininger, M., Kobs, K., Davidson, P., Krause, A. & Hotho, A. Density-based weighting for imbalanced regression. *Machine Learning* **110**, 2187–2211 (2021).

`

93. Ota, K., Oiki, T., Jha, D., Mariyama, T. & Nikovski, D. Can increasing input dimensionality improve deep reinforcement learning? in *International conference on machine learning* 7424–7433 (2020).
94. Lillicrap, T. P. *et al.* Continuous control with deep reinforcement learning. *arXiv preprint arXiv:1509.02971* (2015).
95. Mnih, V. *et al.* Playing atari with deep reinforcement learning. *arXiv preprint arXiv:1312.5602* (2013).
96. Lesort, T., D\'\iaz-Rodr\'\iguez, N., Goudou, J.-F. & Filliat, D. State representation learning for control: An overview. *Neural Networks* **108**, 379–392 (2018).
97. Munk, J., Kober, J. & Babuška, R. Learning state representation for deep actor-critic control. in *2016 IEEE 55th Conference on Decision and Control (CDC)* 4667–4673 (2016).
98. Pathak, D., Agrawal, P., Efros, A. A. & Darrell, T. Curiosity-driven Exploration by Self-supervised Prediction. *34th International Conference on Machine Learning, ICML 2017* **6**, 4261–4270 (2017).
99. Bellemare, M. G. *et al.* Unifying Count-Based Exploration and Intrinsic Motivation. *Advances in Neural Information Processing Systems* 1479–1487 (2016).
100. Lopes, M., Lang, T., Fu, M. T., Germany, B. & Oudeyer, P.-Y. Exploration in Model-based Reinforcement Learning by Empirically Estimating Learning Progress. *Advances in Neural Information Processing Systems* **25**, (2012).
101. Poupart, P., Vlassis, N., Hoey, J. & Regan, K. An analytic solution to discrete bayesian reinforcement learning. *ACM International Conference Proceeding Series* **148**, 697–704 (2006).
102. Liu, M., Zhu, M. & Zhang, W. Goal-Conditioned Reinforcement Learning: Problems and Solutions. *IJCAI International Joint Conference on Artificial Intelligence* 5502–5511 (2022) doi:10.24963/ijcai.2022/770.
103. Vilalta, R. & Drissi, Y. A perspective view and survey of meta-learning. *Artificial Intelligence Review* **18**, 77–95 (2002).
104. Kaelbling, L. P., Littman, M. L. & Moore, A. W. Reinforcement Learning: A Survey. *Journal of Artificial Intelligence Research* **4**, 237–285 (1996).
105. Andrychowicz, M. *et al.* Hindsight Experience Replay. *Advances in Neural Information Processing Systems* **30**, (2017).
106. Yin, X. *et al.* ReRoGCRL: Representation-based Robustness in Goal-Conditioned Reinforcement Learning. (2024).
107. Schulman, J., Wolski, F., Dhariwal, P., Radford, A. & Klimov, O. Proximal policy optimization algorithms. *arXiv preprint arXiv:1707.06347* (2017).
108. Gupta, S. *et al.* In Silico Approach for Predicting Toxicity of Peptides and Proteins. *PLOS ONE* **8**, e73957 (2013).
